# Whole‐genome survey reveals extensive variation in genetic diversity and inbreeding levels among peregrine falcon subspecies

**DOI:** 10.1002/ece3.10347

**Published:** 2023-07-20

**Authors:** Jeff A. Johnson, Giridhar Athrey, Clifford M. Anderson, Douglas A. Bell, Andrew Dixon, Yoshinori Kumazawa, Tom Maechtle, Garrett W. Meeks, David Mindell, Keiya Nakajima, Ben Novak, Sandra Talbot, Clayton White, Xiangjiang Zhan

**Affiliations:** ^1^ Department of Biological Sciences University of North Texas Denton Texas USA; ^2^ Wolf Creek Operating Foundation Wolf Wyoming USA; ^3^ Department of Poultry Science & Faculty of Ecology and Evolutionary Biology Texas A&M University College Station Texas USA; ^4^ Falcon Research Group Bow Washington USA; ^5^ East Bay Regional Park District Oakland California USA; ^6^ California Academy of Sciences San Francisco California USA; ^7^ The Mohamed Bin Zayed Raptor Conservation Fund Abu Dhabi United Arab Emirates; ^8^ International Wildlife Consultants Carmarthen UK; ^9^ Research Center for Biological Diversity Nagoya City University Nagoya Japan; ^10^ Bighorn Environmental Sheridan Wyoming USA; ^11^ Museum of Vertebrate Zoology University of California, Berkeley Berkeley California USA; ^12^ The Japan Falconiformes Center Owariasahi Japan; ^13^ Revive & Restore Sausalito California USA; ^14^ Far Northwestern Institute of Art and Science Anchorage Alaska USA; ^15^ Brigham Young University Provo Utah USA; ^16^ Chinese Academy of Sciences Beijing China

**Keywords:** effective population size, genomic diversity, inbreeding coefficient, life‐history, mutation load, runs of homozygosity

## Abstract

In efforts to prevent extinction, resource managers are often tasked with increasing genetic diversity in a population of concern to prevent inbreeding depression or improve adaptive potential in a changing environment. The assumption that all small populations require measures to increase their genetic diversity may be unwarranted, and limited resources for conservation may be better utilized elsewhere. We test this assumption in a case study focused on the peregrine falcon (*Falco peregrinus*), a cosmopolitan circumpolar species with 19 named subspecies. We used whole‐genome resequencing to generate over two million single nucleotide polymorphisms (SNPs) from multiple individuals of all peregrine falcon subspecies. Our analyses revealed extensive variation among subspecies, with many island‐restricted and nonmigratory populations possessing lower overall genomic diversity, elevated inbreeding coefficients (*F*_ROH_)—among the highest reported, and extensive runs of homozygosity (ROH) compared to mainland and migratory populations. Similarly, the majority of subspecies that are either nonmigratory or restricted to islands show a much longer history of low effective population size (*N*_e_). While mutational load analyses indicated an increased proportion of homozygous‐derived deleterious variants (i.e., drift load) among nonmigrant and island populations compared to those that are migrant or reside on the mainland, no significant differences in the proportion of heterozygous deleterious variants (i.e., inbreeding load) was observed. Our results provide evidence that high levels of inbreeding may not be an existential threat for some populations or taxa. Additional factors such as the timing and severity of population declines are important to consider in management decisions about extinction potential.

## Graphical abstract

By exploring genomic diversity among all named peregrine falcon subspecies, we identified a pattern showing reduced diversity and higher inbreeding levels and runs of homozygosity among island and nonmigratory compared to mainland and migratory subspecies. These patterns are consistent with the effects of long‐term small population size among island and nonmigratory populations, which was further supported by PSMC analyses. Additional analyses focused on mutational load supported these conclusions, and further highlights that high inbreeding levels may not necessarily be of immediate concern and that other factors such as habitat loss and other human‐related activities may be of greater importance in influencing population persistence depending on its demographic history.

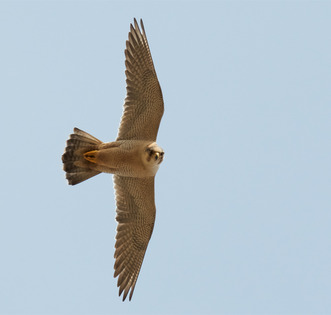

## 1 INTRODUCTION

Intraspecific levels of genetic diversity are often used as a metric for approximating the viability of a species or population and its survival or adaptive potential, with the assumption that those possessing lower diversity are less resilient (Foden et al., 2013). While there is empirical support for that generalized pattern for some cases (DeWoody et al., 2021), the use of such a metric in that way assumes that all species or populations are similar in their potential for standing genetic variation and its relationship with viability over time (see also García‐Dorado & Caballero, 2021; Hoffmann et al., 2017; Teixeira & Huber, 2021). We know, however, that genetic diversity levels can vary considerably depending on a population's demographic history given that effective population size positively correlates with genetic diversity (Kimura, 1983; Wright, 1931) and deleterious genetic variation (Charlesworth & Charlesworth, 1987) in populations at mutation‐drift‐selection equilibrium.

While smaller isolated populations of the same or closely related species are generally expected to have lower genetic diversity compared to larger panmictic populations (Ellegren & Galtier, 2016; Leffler et al., 2012), this does not always correlate to a population's conservation potential. The real‐world results of saving species from extreme bottlenecks has shown that augmenting low genetic diversity has been a successful strategy in almost every application (Frankham, 2015; Ralls et al., 2020); however, recovery from low numbers without genetic diversity augmentation has also been achieved, particularly among island populations (Wiedenfeld et al., 2021). Expected differences in genetic diversity levels based on a species' or population's demographic history should therefore inform recommendations for conservation purposes, and are critical when determining and prioritizing appropriate management, such as promoting habitat protection or increasing population size through augmentation (i.e., genetic rescue). For example, the development of measures to increase population size may not be the best approach if the target population or species has existed at small effective population size for thousands of generations. A more practical approach might be to first identify the specific factors that limit a population or species in the wild, including habitat requirements and quality (Johnson et al., 2009; Razafimanjato et al., 2014; Robinson et al., 2017) and minimize human–wildlife conflict and persecution (Nyhus, 2016). Nevertheless, developing captive breeding strategies for eventual or immediate release may provide an insurance mechanism for rescuing target populations of extremely small size with a high probability of extirpation, at least until informed decisions can be made concerning conservation actions (Farhadinia et al., 2020; Gooley et al., 2017; Ishtiaq et al., 2015).

Because increasing levels of genetic diversity are often seen as an important goal for sustaining species diversity (Frankham, 2015; Hoban et al., 2021; Laikre et al., 2020; Ralls et al., 2020) and predicting resiliency in the face of changing climates (Foden et al., 2013), more studies are needed to quantify to what degree genomic diversity varies among and within species characterized by different long‐term effective population sizes. This will facilitate our ability to distinguish whether levels of genomic diversity within a target population or lineage are likely to provide the necessary resilience or identify populations or lineages of increasing conservation concern. Whole‐genome sequencing provides for cost‐effective generation of DNA sequence data, and the number of studies quantifying whole‐genome diversity levels within and among species have increased (Bravo et al., 2021). This has provided increased opportunity to examine how genetic diversity varies among and within taxa.

In addition to a species' demographic history, its life history can also influence patterns of genomic diversity and should be considered when justifying specific management decisions. For example, species with large geographic distributions with subspecies that possess strikingly different life histories (e.g., migrant vs. nonmigrant) or occupy areas with differing spatial constraints (e.g., mainland vs. island) may have differences in their standing genetic variation that can influence population persistence differently. Further, to what degree low genome‐wide diversity and high inbreeding levels corresponds with increased mutational load (i.e., deleterious variants within coding regions) is an important consideration because a generalized measure of genetic diversity may not provide the necessary information to infer a populations' level of fitness or likelihood of experiencing inbreeding depression (Bertorelle et al., 2022; Caballero et al., 2017; Kardos et al., 2021; Teixeira & Huber, 2021). Larger populations that experience a significant rapid decline in size are more likely to experience an increase in the proportion of deleterious alleles due to random genetic drift, thereby becoming more vulnerable to extinction due to expression of those alleles in a homozygous state (Kyriazis et al., 2021; Smeds & Ellegren, 2022; Van Oosterhout, 2020). In contrast, populations that have persisted at small size for ≥100s generations have had the opportunity to purge their mutational load (or inbreeding load) of both lethal and nonlethal mutations (Caballero et al., 2017; Kardos et al., 2021; Robinson et al., 2022; Van der Valk et al., 2021). Quantifying differences in mutational load based on the measures of loss of function (LOF) can provide additional insight into the apparent risk of extinction due to inbreeding.

**FIGURE 1:**
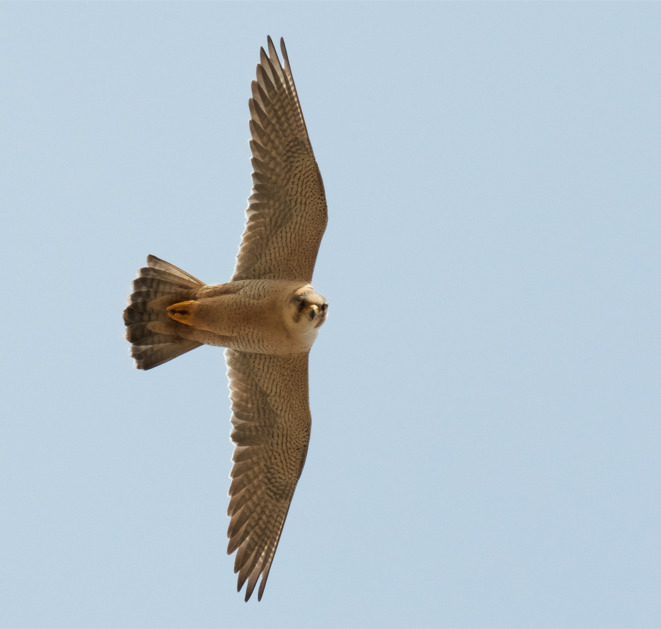
Peregrine falcon (*Falco peregrinus babylonicus*, or Red‐naped Shaheen) flying near its nest in southern Mongolia. Photo taken by Jeff A. Johnson.

**FIGURE 2:**
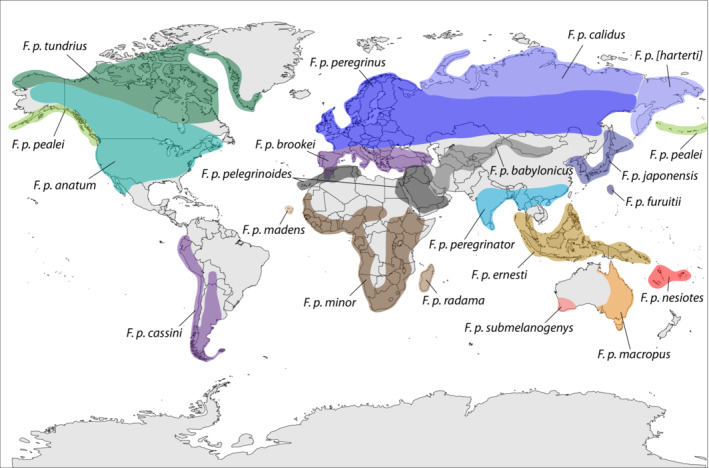
Geographic distribution of peregrine falcon subspecies (adapted from White, Cade, & Enderson, 2013). The exact areas of contact and degree of overlap are largely unknown among many neighboring subspecies. Some authorities do not recognize *F. p*. [*harterti*] as a valid subspecies and subsume with *F. p. japonensis*.

At least 19 subspecies of peregrine falcon (*Falco peregrinus*; Figure 1) are currently named (White, Cade, & Enderson, 2013), many of which possess differing life‐history strategies and capacity for long‐distance dispersal despite their relatively recent common ancestry within the past one million years (Fuchs et al., 2015). Peregrine subspecies are distributed throughout the world (Figure 2) with variation in migratory behavior and differing overall geographic ranges (i.e., population size). For example, multiple subspecies are year‐round island residents (*F. p. nesiotes*, *F. p. madens*, and *F. p. radama*), and others include long‐distance migrants with large geographic distributions throughout the Arctic during their breeding season (*F. p. tundrius*, *F. p. peregrinus*, *F. p. calidus*). In addition, multiple subspecies possess both island and mainland populations (*F. p. pealei*, *F. p. cassini*, *F. p. peregrinator*, *F. p. ernesti*) or varying levels of migratory behavior depending on local environmental conditions and resource availability (*F. p. anatum*, *F. p. peregrinus*, *F. p. babylonicus*; see Table S1; Gu et al., 2021; White, Cade, & Enderson, 2013). Although much genetic research has examined levels of diversity using a restricted set of presumably neutral or functional loci among peregrine falcon populations and subspecies (Bell et al., 2014; Brown et al., 2007; Gangoso et al., 2012; Jacobsen et al., 2008; Johnson et al., 2010; Ponnikas et al., 2017; Sonsthagen et al., 2022; Talbot et al., 2011, 2017; Weaving et al., 2021; White, Sonsthagen, et al., 2013), it remains unclear to what degree genomic diversity varies within the species on a broad geographic scale and whether the observed patterns are expected due to life history differences or spatial constraints that may have developed since subspecies and population differentiation.

Using whole‐genome resequencing data from all 19 named peregrine falcon subspecies, we compared genomic diversity levels among subspecies to investigate differences in life‐history and spatial constraints. For example, is genomic diversity lower among populations that are nonmigratory or reside on islands compared to migratory and mainland populations? Further, is there evidence to suggest those differences may influence fitness in terms of mutational load, and should this be a concern from a conservation perspective? These results can provide a useful model that can inform resource managers on how genomic diversity levels can vary within and among conspecific subspecies, and whether standing genetic variation among most peregrine falcon subspecies and their populations is a reflection of long‐term demographic history rather than recent changes in population size.

## 2 METHODS

### 2.1 Sampling and whole‐genome resequencing

A total of 56 blood samples and seven toe‐pad tissues were obtained from wild caught peregrine falcons or museum specimens, respectively (Table S1). No samples were used in this study from geographic areas in the midwestern and eastern United States or in Germany where captive‐reared individuals of potential mixed subspecies ancestry were released intentionally for conservation‐related purposes (Burnham & Cade, 2003; Saar, 1988). We extracted genomic DNA from contemporary samples using standard methods in multiple laboratories among collaborators. Museum toe‐pads were processed at the UCSC Paleogenomics Lab in a room designated for work with only historic specimens to prevent potential cross contamination with modern tissue samples. Clean suits, facial masks, and sterile gloves were worn at all times when handling historic specimens, as per the standard protocols for working with ancient DNA (Cooper & Poinar, 2000). DNA from toe‐pads was extracted using the Qiagen Blood & Tissue Kit with ancient DNA‐specific modifications (Fulton et al., 2012).

The majority of raw sequence reads from the contemporary samples (*n* = 54) were generated from libraries prepared using the Illumina Nextera DNA Flex Library Preparation Kit and sequenced at ~10× average depth of coverage on two S4 150PE Flow Cell lanes on an Illumina NovaSeq 6000 platform at North Texas Genome Center at the University of Texas in Arlington. Sequence reads for *F. p. japonensis* (*n* = 2) were prepared using New England BioLabs Next Ultra DNA Library Prep Kit and generated (~4.5× depth) on an Illumina MiSeq platform using the MiSeq Reagent Kit v3 at Nagoya City University, Japan, while libraries for seven museum samples (*F. p. furuitii*, *n* = 2; *F. p. ernesti*, *n* = 3; *F. p. peregrinator*, *n* = 2) were prepared following Meyer and Kircher (2010) and then sequenced at 9–15× depth on a single S4 150PE Flow Cell lane on an Illumina NovaSeq 6000 platform at North Texas Genome Center.

To obtain whole‐genome sequence data from all 19 peregrine falcon subspecies, additional raw sequence reads for *F. p. peregrinus* (*n* = 14) and *F. p. calidus* (*n* = 9) were obtained from GenBank (accession number PRJNA686418), including a subset of samples identified as “*F. p*. [*harterti*]” (*n* = 8) from northeast Russia, which has been grouped with *F. p. calidus* or *F. p. japonensis* depending on the authority (see Dixon et al., 2012; White, Cade, & Enderson, 2013). These raw sequence reads had between 28 and 35× average depth of coverage (Table S1; see also Gu et al., 2021). Raw sequence reads from three outgroup taxa were obtained either from NCBI SRA (Saker Falcon, *F. cherrug*, SRR516282; Prairie Falcon, *F. mexicanus*, SRR6807233) or generated as described above (Taita Falcon, *F. fasciinucha*).

All raw sequence reads were processed using standard bioinformatic tools and filtering criteria when working with contemporary and museum sample whole‐genome resequencing data. Specifically, adapters were removed using SeqPrep (https://github.com/jstjohn/SeqPrep) and Cutadapt (Martin, 2011) with a minimum Phred quality score of 20. SeqPrep was also used with default settings to merge overlapping paired‐end reads for the museum samples to increase fragment length and improve mapping efficiency. Trimmed reads were then mapped to a peregrine falcon chromosomal assembly (falPer2; GenBank assembly accession: GCA_001887755.1; Joseph et al., 2018) using BWA‐MEM (Li, 2013) or BWA‐ALN (Li & Durbin, 2009) v.0.7.17 for the contemporary and museum samples, respectively. Default parameters were used for mapping the contemporary samples, and deactivated seeding (−l 16,500), allowing for more substitutions (−n 0.01) and up to two gaps (−o 2) were included to improve mapping of the museum samples (Schubert et al., 2012). Following initial processing, two samples (*F. p. anatum* and *F. p. macropus*) had low average depth of coverage (<3×) and were excluded from further analysis.

All generated BAM files from the merged museum sample files were processed using mapDamage v.2.1.1 (Jónsson et al., 2013) to verify nucleotide misincorporation patterns typical of degraded DNA (Figure S1). The first 5 bp from the 5′ end of each PE sequence read were then trimmed using FASTQ Trimmer (http://hannonlab.cshl.edu/fastx_toolkit/), remapped to the reference genome using BWA‐ALN, and base quality scores were rescaled using mapDamage. The rescaled BAM files generated for the museum samples were used in all subsequent analyses.

Following the removal of PCR duplicates using Samtools v.1.6 (Li, 2011) and the addition of read group information using Picard tools v2.10 (http://broadinstitute.github.io/picard), mapped reads were realigned to minimize mismatched bases using RealignerTargetCreator and IndelRealigner with GATK v.3.8 (McKenna et al., 2010). Variant calling was then performed using GATK HaplotypeCaller (in GVCF mode) and GenotypeGVCFs with default parameters (Poplin et al., 2018). Raw SNPs were then filtered using BCFtools v.1.6 (Danecek et al., 2021) and custom scripts for quality and depth using recommended GATK hard filters (QD < 2.0 || FS > 60.0 || MQ < 40.0 || MQRankSum < −12.5 || ReadPosRankSum < −8.0 || SOR > 3 || DP < 3), with both indels and SNPs within five base pairs removed. We also excluded sites with heterozygosity >60% (to remove possible paralogs) and missingness above 10% among all samples and required heterozygous genotypes to have an allele balance of 20%–80%.

### 2.2 Annotated assembly

We annotated the falPer2 genome assembly using a total of 31,142 peregrine falcon coding sequences deposited in GenBank prior to January 18, 2020. These coding sequences were used as evidence for annotating the genome assembly using the software Maker v.3 (Cantarel et al., 2008). First, we ran the annotation pipeline using the AUGUSTUS software for ab initio prediction (Stanke et al., 2006) using the domestic chicken as transcripts. The 31,142 coding sequences were provided as EST evidence. We used the Maker‐generated annotations to perform a second round of annotations to improve the quality. We evaluated the resulting annotation using the AED scores (annotation edit distance). The second round of annotations returned 10,388 genes with over 95% of them with an AED < 0.5. The identified genes were further annotated with information about protein family (based on blastp searches against the UNIPROT and SWISSPROT protein databases) and putative function (based on gene ontology hits using InterProScan).

Prior to further analysis, we partitioned the resulting filtered SNP dataset into coding (genic) and noncoding (intergenic) VCF files using the functionally annotated assembly and then separated into autosomal (chromosomes 1–18) and Z chromosome datasets. SNPs were then pruned for linkage disequilibrium for each dataset using BCFtools v.1.6 (Danecek et al., 2021) with *r* ^2^ set to 0.6 and a window size of 1000 bp. Unless stated otherwise, we used biallelic SNPs from chromosomes 1 to 18 (i.e., Z and unplaced scaffolds excluded) partitioned as intergenic dataset (2,346,373 SNPs).

### 2.3 Subspecies verification

We generated a NeighborNet network using SplitsTree4 (Hudson et al., 2008) to verify clustering of individuals by subspecies and population. The NeighborNet network was generated using the autosomal intergenic biallelic SNP dataset with the GTR substitution model and rate matrix (rmat = 1.0120 5.8251 1.0125 0.7695 5.8966) values from jModelTest v.2.1.10 (Darriba et al., 2012), which was identified as the best model based on the Bayesian information criterion (BIC) assuming five substitution schemes, unequal base frequencies, no rate variation among sites and the “best” tree search strategy. Splits with weight below 5 × 10^−4^ threshold were filtered to simplify the network and reduce the number of branches. A total of 191 of 341 splits were retained with 98.7% proportional weight remaining after filtering.

A species tree was also reconstructed under the coalescent module using SVDquartets (SVDQ; Chifman & Kubatko, 2014). This species tree method has been shown to be robust to gene flow between closely related taxa (Long & Kubatko, 2018). Based on preliminary or previous analyses (Talbot et al., 2017) that suggested differentiation between some mainland and/or island populations, individuals from the following six subspecies were grouped by population: *F. p. nesiotes* (Fiji and Vanuatu), *F. p. pelegrinoides* (Canary Islands and Israel), *F. p. anatum* (Alaska/Canada and western United States), *F. p. pealei* (Aleutian Islands and SE Alaska), *F. p. cassini* (Falkland Islands and Chile), and *F. p. peregrinator* (southeast China, India and Sri Lanka). All remaining individual samples were grouped according to subspecies.

The SVDQ analyses were run as implemented in PAUP* v.4.0a (build 168; Swafford, 2003) in the “species tree” mode with exhaustive quartet sampling using the QFM algorithm. We excluded variants with missing calls among samples prior to analysis resulting in a total of 206,863 biallelic autosomal SNPs. The species tree analysis included 100 bootstrap replicates to assess branch support among the sampled taxonomic groups, and rooted using three outgroup taxa (saker falcon, prairie falcon, and Taita falcon).

### 2.4 Genomic diversity

To compare diversity measures between subspecies, including differences in life‐history traits (migratory vs. nonmigratory) and spatial distributions (island vs. mainland), we calculated the inbreeding coefficient and the proportion of SNPs that were heterozygous for each sample. The proportion of total SNPs identified as heterozygous for each sample was calculated using BCFtools v1.6, and individual inbreeding coefficients (*F*_UNI_) were calculated based on the correlation between uniting gametes using Genome‐wide Complex Trait Analysis v.1.93.0 (GCTA; Yang et al., 2011).

Local estimates of runs of homozygosity (ROH) were calculated for each sample using BCFtools with option ‐G 30 and the filtered autosomal dataset including chromosomes 1–18 and both genic and intergenic regions. We then estimated F_ROH_ as the overall proportion of each genome with ROH segments ≥100 kb and ≥2 Mb based on the summed ROH length of a generated pseudo‐genome with 100% homozygous genotypes (987,758,487 bp; see Robinson et al., 2022), with differences in each segment size group providing insight on the relative recency of inbreeding within the population. In general, smaller populations have more and longer ROH segments (>2 Mb) than larger populations, especially those experiencing recent mating between related individuals (Ceballos et al., 2018). However, long ROH segments will break down over time due to recombination, and an increasing proportion of smaller ROH segments (>100 kb) are likely in populations with long‐term small population size. Populations maintained at small population size for many generations that have also experienced recent inbreeding should possess both small and long ROH segments (Ceballos et al., 2018; Pemberton et al., 2012). The two‐tailed Mann–Whitney *U* test was used to compare diversity measures between island versus mainland and migratory versus nonmigratory subspecies. Subspecies that can be identified as both migratory and nonmigratory were excluded from the comparison to avoid any uncertainty in grouping samples.

### 2.5 Mutational load estimation

To determine the effects of the SNP variants found across falcon population samples, we performed effect prediction using SnpEff v.4 (Cingolani et al., 2012). We first generated a custom genome database for the peregrine using the falPer2 genome assembly and the annotation GTF generated in this study. Following this, we passed each filtered variant file through SnpEff to generate an annotated list of genes impacted by SNP variants. We considered two categories of variants in this analysis. First, we subset the ‘HIGH Impact Variants’ (HIV) as defined in SnpEff, which are assumed to have a highly disruptive impact on the product of the gene, including but not limited to nonsense mutations, and predicted to cause deleterious gene effects. Second, we separately considered LOF mutations, which only included Stop Gain and Stop Loss mutations. These two lists are not always mutually inclusive but provide a measure of variants that may lead to functional changes and thus, possible deleterious mutations.

Each individual was then categorized according to genotype as either homozygous‐derived when compared to the reference or heterozygous‐derived within each impact group. This was done to investigate patterns associated with the contrasting effects of purifying selection and genetic drift and their potential influence on the proportion of each variant observed within populations or subspecies depending on their respective long‐term *N*_e_. Within populations that have persisted at small *N*_e_, theory predicts fewer HIGH Impact and LOF mutations as heterozygotes (i.e., reduced inbreeding load) because of the increased likelihood of inbreeding and the elimination of recessive variants by purifying selection when exposed as homozygotes (Robinson et al., 2022), yet their fixation as homozygotes is also likely due to random genetic drift (i.e., increased drift load; Henn et al., 2015; Van Oosterhout, 2020). In contrast, deleterious mutations are more likely to accumulate as heterozygotes in large populations (i.e., increased inbreeding load) because they are less likely to be exposed as homozygotes due to less frequent inbreeding, and random genetic drift is less likely to influence their frequency or potential fixation within the population (i.e., reduced drift load; see also Robinson et al., 2023).

We normalized each measure by dividing by the total number of called genotypes observed for each individual. This helped control for differences in depth of coverage and missing genotypes among samples. Similar to the diversity measure comparisons, we used the two‐tailed Mann–Whitney *U* tests to compare homozygous and heterozygous levels of both HIGH Impact and LOF variants between island versus mainland and migratory versus nonmigratory subspecies or populations. This tested whether there were differences in the number of each variant type (i.e., HIGH Impact and LOF) between each grouping category under the assumption that a higher homozygous value equates to a comparably higher drift load, whereas a lower heterozygous value represents a correspondingly lower inbreeding load, or a reduced risk of future inbreeding depression, as expected for small long‐term *N*_e_.

### 2.6 Demographic history

A pairwise sequentially Markovian coalescent (PSMC v.0.6c.5; Li & Durbin, 2011) model was used to estimate temporal changes in effective population sizes (*N*_e_) among sampled subspecies. Consensus sequences were generated for autosomal chromosomes 1–18 using the previously filtered VCF datafiles including both genic and intergenic biallelic sites using GATK v.3.8 FastaAlternateReferenceMaker. The number of iterations (*N*) was set to 25, *t* (*T*_max_) was 15 and p (atomic time interval) was 64 (4 + 25*2 + 4 + 6) with 100 bootstrap replicates for each run. We used the mutation rate of 1.65 × 10^−9^ per year and a generation time of 6 years (Zhan et al., 2013).

For comparison purposes, we calculated the long‐term mean *N*_e_ for each sampled individual over the entire time period analyzed using PSMC (approximately 1 Myr). Because the accuracy of PSMC for estimating *N*_e_ for the most recent time points is low, often resulting in a noisy signal due to a lower number of coalescent events, we excluded the first four most recent time points from our calculations of mean *N*_e_ for each sampled individual (Brüniche‐Olsen et al., 2021; Leroy et al., 2021). Linear regression was then used to identify correlations between diversity measures and long‐term estimates of *N*_e_.

## 3 RESULTS

### 3.1 Phylogenetic analysis

**FIGURE 3:**
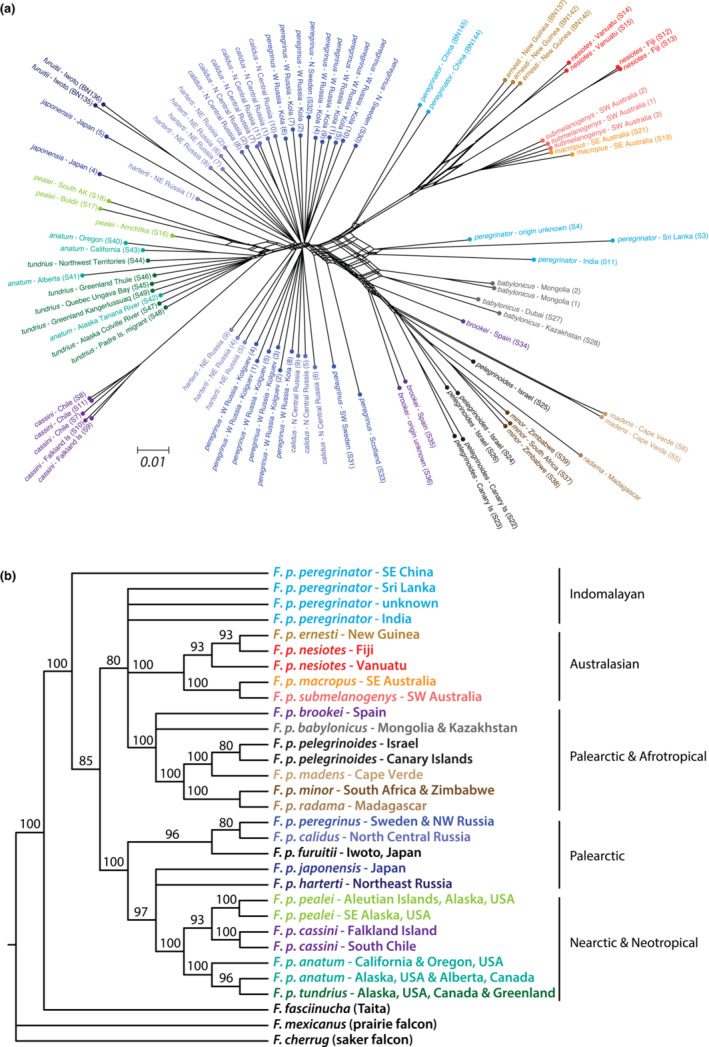
Phylogenetic relationships among peregrine falcon subspecies based on autosomal intergenic biallelic SNPs. (a) NeighborNet network with scale bar indicating 1% divergence. (b) SVD_quartets_ species tree excluding sites with missing calls. Values at nodes represent 100 bootstrap support values with nodes possessing <80 bootstrap support collapsed to form a polytomy. Sample names are color coded by subspecies with ID labels in network as provided in Table S1.

Based on whole‐genome resequencing of multiple individuals representing all 19 named peregrine subspecies, the majority of samples clustered according to their taxonomy as shown in the NeighborNet network (Figure 3a). The few exceptions include a grouping of *F. p. peregrinus* and *F. p. calidus* samples forming a broad reticulation in the center of the network, and *F. p. anatum* samples collected in the northern extent of their distribution in Alaska and Alberta clustering among *F. p. tundrius* samples. The remaining *F. p. anatum* samples from California and Oregon formed a separate cluster. Of the four subspecies with more than one sample that include both mainland and island populations, individuals from *F. p. pealei*, *F. p. cassini* and *F. p. pelegrinoides* clustered with either their mainland or their island counterparts. The *F. p. peregrinator* samples from China formed a cluster separate from the other *F. p. peregrinator* samples from India and Sri Lanka (Figure 3a).

The species tree provided similar results highlighting low resolution at the base of the tree, but subspecies grouped within clades that corresponded largely with broad geographic regional distributions (Figure 3b; i.e., Australasian, Palearctic, Nearctic, Afrotropical). Within those clades, sister relationships based on geographic proximity were observed, and sampled subspecies with multiple subpopulations were largely sister to each other. The two exceptions were the southern *F. p. anatum* population sister to a clade including both northern *F. p. anatum* and *F. p. tundrius*, and a lack of resolution supporting definitive sister relationship between *F. p. peregrinator* samples from China and those collected in India and Sri Lanka (Figure 3b).

### 3.2 Patterns of genome‐wide diversity

**FIGURE 4:**
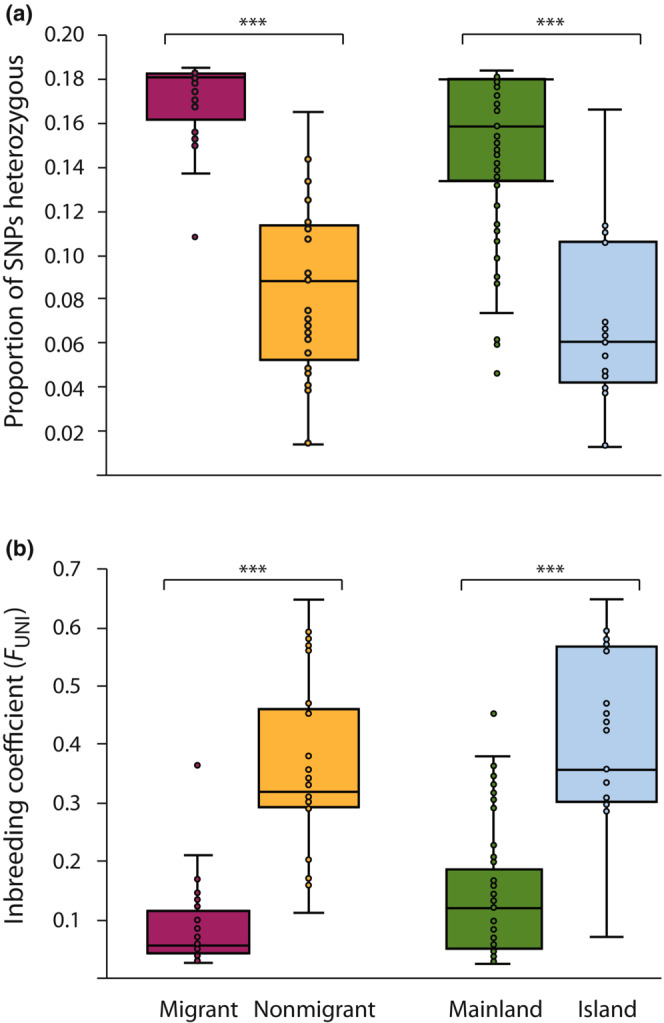
Proportion of SNPs (a) heterozygous and (b) individual inbreeding coefficients (*F*_UNI_) for peregrine falcons grouped as migrants (*n* = 45) or nonmigrants (*n* = 29), and mainland (*n* = 68) or island (*n* = 22) populations using autosomal intergenic biallelic SNPs. Horizontal lines within boxplots and bounds of boxes/whiskers represent medians and standard quartile ranges, respectively (Mann–Whitney two‐tailed *U* test, ***p < .0001). See Table S1 for subspecies classification.

**FIGURE 5:**
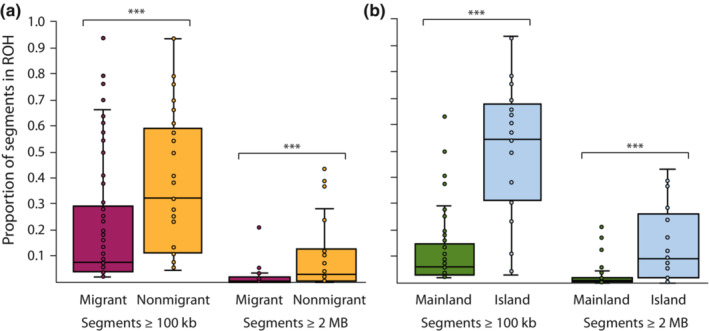
Proportion of genomic segments in ROH ≥100 kb and ≥2 Mb in size among (a) migrant or nonmigrant and (b) mainland or island peregrine falcon populations using autosomal genic and intergenic SNPs. Horizontal lines within boxplots and bounds of boxes/whiskers represent medians and standard quartile ranges, respectively (Mann–Whitney two‐tailed *U* test, ***p < .0001). See Table S1 for subspecies classification.

Genome‐diversity measures were consistently lower among both island and nonmigratory groupings of subspecies compared to mainland and migratory groups, respectively (Figures 4, 5; see also Figures 2s, 3s). The proportion of autosomal SNPs identified as heterozygous ranged from 0.029 ± 0.015 (mean ± SD, *n* = 2) to 0.181 ± 0.003 (*n* = 9) in *F. p. nesiotes* and *F. p. calidus*, respectively (Figure S2a), with average individual estimates from island (*n* = 21, 9 subspecies) and nonmigratory (*n* = 29, 10 subspecies) populations significantly lower than mainland (*n* = 69, 14 subspecies) and migratory (*n* = 45, 5 subspecies) populations (Figure 4a; Mann–Whitney *U* test, *Z* = 5.83, *p* < .0001; *Z* = 6.98, *p* < .0001, respectively). A similar pattern was observed with individual inbreeding coefficients (*F*_UNI_), which ranged from 0.044 ± 0.012 (*n* = 9) to 0.647 (*n* = 1) in *F. p. calidus* and *F. p. radama*, respectively (Figure S2b), with island and nonmigratory populations having significantly higher individual average *F*_UNI_ values than mainland and migratory populations (Figure 4b; *Z* = 5.54, *p* < .0001; *Z* = 6.81, *p* < .0001, respectively).

Variation in heterozygosity within subspecies was also observed with a few having noticeably high standard deviations among samples inhabiting island (e.g., *F. p. pealei* and *F. p. nesiotes*) and mainland (e.g., *F. p. anatum* and *F. p. peregrinator*) populations (see Figure S2a). The same was observed with inbreeding coefficient estimates (*F*_UNI_; Figure S2b) with samples possessing high heterozygosity correspondingly having low *F*_UNI_. Among the four subspecies that had both island and mainland sampled populations (*F. p. pealei*, *F. p. cassini*, *F. p. peregrinator*, and *F. p. pelegrinoides*), the island populations generally had lower heterozygosity and higher inbreeding coefficients (Figure S2). The exception was with *F. p. pealei*, where high variability between the two island samples resulted in similar diversity measures between the two groups.

Island and nonmigratory populations also possessed a higher proportion of segments identified as ROH compared to mainland and migratory populations for segments ≥100 kb (*Z* = 5.46, *p* < .0001; *Z* = 7.10, *p* < .0001, respectively) and ≥2 Mb (*Z* = 4.32, *p* < .0001, *Z* = 3.42, *p* < .001; Figure 5). Variation among individuals among subspecies and populations was also high with some species showing significant variation between populations not only with the proportion of ROH segments ≥100 kb but also with overall proportion ≥ 2 Mb suggesting differences in the timing and magnitude of population decline may exist (e.g., *F. p. nesiotes*; Figure S3).

### 3.3 Mutation load comparisons

**FIGURE 6:**
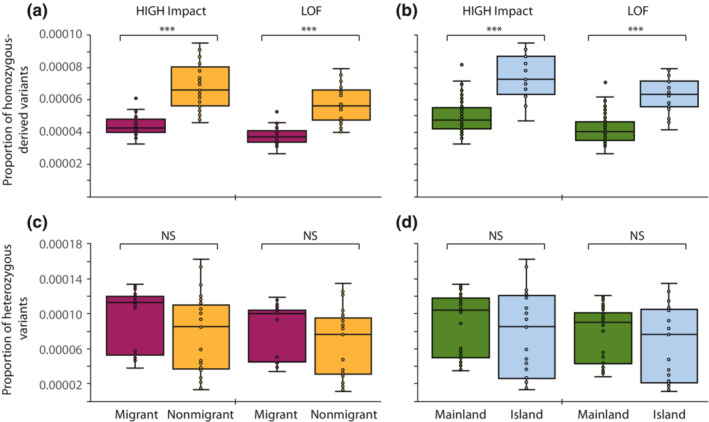
Proportion of homozygous‐derived and heterozygous HIGH Impact and LOF variants among (a, c) migrant and nonmigrant and (b, d) mainland and island peregrine falcon populations. Horizontal lines within boxplots and bounds of boxes/whiskers represent medians and standard quartile ranges, respectively (Mann–Whitney two‐tailed *U* test, NS = not significant, Bonferroni‐adjusted *p*‐value > .006; ***p < .0001). See Table S1 for subspecies classification.

Variation in both homozygous‐ and heterozygous‐derived variants identified as either HIGH Impact or LOF was observed among subspecies (Figure S4). In general, individuals identified as belonging to either migrant or mainland populations possessed on average lower homozygous‐derived HIGH Impact and LOF genotypes (i.e., lower drift load) compared to nonmigrant (*Z* = 6.78, *p* < .0001; *Z* = 6.43, *p* < .0001, respectively) or island populations (*Z* = 5.91, *p* < .0001; *Z* = 5.96, *p* < .0001, respectively; Figure 6a,b). In contrast, however, a comparable proportion of heterozygous HIGH Impact and LOF variants (i.e., inbreeding load) was observed among migrants compared to nonmigrants (*Z* = 2.46, *p* = .014; *Z* = 2.61, *p* = .009, respectively) and among island and mainland populations (*Z* = 1.23, *p* = .219; *Z* = 1.29, *p* = .197, respectively; Figure 6c,d) after adjusting significance due to multiple simultaneous comparisons (Bonferroni‐adjusted *p*‐value = .006).

### 3.4 Demographic history

**FIGURE 7:**
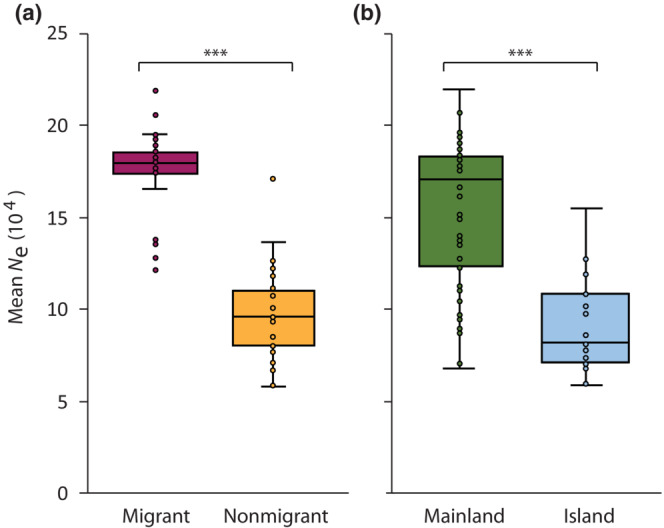
Mean effective population size (*N*_e_) over the past ~1 million years for (a) migrant and nonmigrant and (b) mainland and island peregrine falcon populations based on autosomal SNPs using PSMC. Horizontal lines within boxplots and bounds of boxes/whiskers represent medians and standard quartile ranges, respectively (Mann–Whitney two‐tailed *U* test, ***p < .0001). See Table S1 for subspecies classification.

Based on PSMC, island and nonmigratory peregrine subspecies consistently had lower mean *N*_e_ over the last one million years compared to mainland (*Z* = 5.37, *p* < .0001) and migratory (*Z* = 6.92, *p* < .0001) subspecies, respectively (Figure 7; see also Figure S5). These same patterns were also largely correlated with estimates of genomic diversity. Mean *N*_e_ for each sampled individual was positively correlated with heterozygosity (*R* ^2^ = .846, *F*_1,89_ = 493.9, *p* < .0001) and negatively correlated with both *F*_UNI_ (*R* ^2^ = .735, *F*_1,89_ = 251.2, *p* < .0001) and proportion of ROH segments ≥100 kb (*R* ^2^ = .570, *F*_1,89_ = 117.8, *p* < .0001) and ≥2 Mb (*R* ^2^ = .174, *F*_1,89_ = 18.8, *p* < .0001; Figure S6).

Comparatively similar results were observed with our mutation load results showing a relatively weak but significant positive correlation between *N*_e_ and the proportion of heterozygous HIGH Impact and LOF genotypes (i.e., inbreeding load) for individuals belonging to either migrant or nonmigrant (*R* ^2^ = .075, *F*_1,71_ = 5.772, *p* = .0187; *R* ^2^ = .078, *F*_1,71_ = 6.021, *p* = .0166, respectively) and mainland or island (*R* ^2^ = .055, *F*_1,88_ = 5.139, *p* = .0258; *R* ^2^ = .055, *F*_1,88_ = 5.083, *p* = .0266, respectively) populations (Figure S7e–h). We also found a stronger but negative correlation between *N*_e_ and the proportion of homozygous‐derived HIGH Impact and LOF genotypes with individuals from either migrant or nonmigrant (*R* ^2^ = .638, *F*_1,71_ = 125.0, *p* < .0001; *R* ^2^ = .634, *F*_1,71_ = 122.2, *p* < .0001, respectively) and mainland or island (*R* ^2^ = .674, *F*_1,88_ = 181.7, *p* < .0001; *R* ^2^ = .673, *F*_1,88_ = 181.3, *p* < .0001, respectively) populations (Figure S7a–d). However, variation existed within groups, particularly among heterozygous variants, as some subspecies, for example, are shown to possess a much higher proportion of heterozygous HIGH Impact and LOF variants compared to other categorically similar mainland or island subspecies (e.g., *F. p. harterti* vs. *F. p. brookei* or *F. p. japonensis* vs. *F. p. furuitii*) or even between island populations of the same subspecies (e.g., *F. p. nesiotes*; Figure S4c,d).

## 4 DISCUSSION

Significant variation in genomic diversity levels exists among peregrine falcon subspecies with many nonmigratory and island‐restricted populations possessing low heterozygosity, high inbreeding coefficients, and extensive ROHs. In fact, inbreeding coefficients calculated for multiple peregrine subspecies in this study are the highest reported to our knowledge for a wild population based on whole‐genome resequencing data (e.g., *F*_UNI_ = 0.647; *F. p. radama*), far exceeding those obtained from multiple endangered avian and mammalian species reported elsewhere based on the same metric (Feng et al., 2019). Because the peregrine falcon is a relatively young species having diverged from its common ancestor with Taita falcon within the past one million years (Bell et al., 2014; Fuchs et al., 2015), the observed uncertainty with low resolution at the base of the species tree and the broad reticulation at the center of the network were not unexpected given the potential for incomplete lineage sorting among subspecies following their rapid diversification. Yet, sufficient resolution was recovered toward the tips of the species tree with clustering corresponding largely to geographic proximity (Figure 3). Therefore, each subspecies' life‐history characteristics are likely influencing their genomic diversity levels as sampled for this study including the proportions of deleterious mutations in the form of LOF variants specific to each of their unique demographic histories rather than more historic patterns generated prior to subspecies differentiation. As shown here and elsewhere (Dussex et al., 2021; Robinson et al., 2022; Tian et al., 2022), the proportion of deleterious mutations can vary depending on long‐term *N*_e_ with populations at small size often possessing reduced inbreeding load and increased drift load due to the effects of purifying selection and random genetic drift, respectively (see Bertorelle et al., 2022; Robinson et al., 2023).

These results have important implications in how genetic data are used to help prioritize efforts when managing wild populations (Van Oosterhout, 2020). We should not assume that all small, isolated populations are likely to be more susceptible to extinction due to inbreeding, especially if they have been at small size for hundreds and thousands of generations (e.g., island endemics; Kyriazis et al., 2021). Instead, larger populations that have experienced recent and significant population declines (i.e., bottleneck event) are more likely to be of immediate concern due to inbreeding depression from the expression of deleterious alleles (or their inbreeding load) when homozygous (Bertorelle et al., 2022; Bortoluzzi et al., 2020; Lohr & Haag, 2015; Robinson et al., 2019, 2023; Smeds & Ellegren, 2022). In fact, recognition is warranted for the timely efforts instituted in the late 1960s in both North America and Europe to reverse the dramatic declines observed among historically abundant mainland peregrine falcons in North America and Europe due to exposure from manufactured organochlorines including DDT (1,1,1‐trichloro‐2,2‐bis[p‐chlorophenyl]‐ethane) and its metabolites (Cade et al., 1971; Ratcliffe, 1967; see Cade & Burnham, 2003; White et al., 2020). The captive propagation and release of thousands of peregrines aided in the recovery of the species in areas most impacted by DDT by reintroducing or supplementing existing populations with lost genetic diversity (Brown et al., 2007; Jacobsen et al., 2008; Ponnikas et al., 2017).

While the captive propagation and release efforts undoubtedly helped the species recovery in those areas where population size had been most severely impacted, we do not know the extent to which the use of multiple subspecies, specifically in the midwestern and eastern United States (Burnham & Cade, 2003) and in Germany (Saar, 1988; Wink, 2015) may have influenced the genetic composition of extant wild populations (Tordoff & Redig, 2001). Because *F. p. anatum* was essentially extirpated east of the Rocky Mountains with only one wild breeding pair known by the late 1960s in southern Quebec, federal approval was obtained to use individuals regardless of subspecies designation for captive breeding and release of their offspring in the midwestern and eastern United States (Burnham & Cade, 2003). Those subspecies included *F. p. anatum*, *F. p. tundrius*, and *F. p. pealei* from western and northern North America, but also *F. p. peregrinus* and *F. p. brookei* from Europe (Scotland and Spain, respectively), *F. p. cassini* from South America (Chile), and *F. p. macropus* from Australia (Burnham & Cade, 2003). This approach was used intentionally in areas within the United States where peregrines had been completely extirpated to maximize genetic variability among released offspring with the intent to provide the reestablished population with an increased potential for adaption in their novel environment. In western Europe, captive breeding facilities in both Sweden and Germany used the resident subspecies, *F. p. peregrinus* but also included neighboring Mediterranean peregrines, *F. p. brookei*, for breeding purposes to generate offspring in Germany for eventual release (Linberg, 1988; Saar, 1988).

For this study, we intentionally excluded samples from those areas where captive‐reared peregrines of mixed ancestry were released to avoid potential complications due to admixture of multiple subspecies. Similar to previous studies, however, we did observe an apparent lack of differentiation between northern *F. p. anatum* and *F. p. tundrius* individuals (Brown et al., 2007; Johnson et al., 2010; Talbot et al., 2017; White, Sonsthagen, et al., 2013), but we also identified differentiation between southern and northern *F. p. anatum* sampled individuals (Figure 3). To what degree the captive propagation and release programs may have influenced those patterns deserves further study, but at least in those areas where the samples from this study originated, most peregrine subspecies designations were supported, as demonstrated for the first time by the correspondence of clustering of individuals based on their taxonomic identification. Therefore, additional analyses are warranted relative to subspecies comparisons in how each of their respective demographic histories may have influenced their standing genomic diversity.

The approximate timing of population decline relative to its long‐term size has important implications concerning management decisions to minimize the likelihood of local extirpation. For example, inbreeding may not necessarily result in inbreeding depression or the reduction in fitness among offspring produced by inbred individuals (see references in Kardos et al., 2021). As populations decline in size, the probability of mating among relatives increases. Inbreeding depression results from the expression of largely recessive deleterious alleles, which is more likely to occur when present among breeding relatives because their offspring will possess a higher frequency of homozygous genotypes compared to a population with randomly bred individuals (Charlesworth & Willis, 2009). The magnitude of inbreeding depression will depend on the number of deleterious alleles that exist within a population, that is, its genetic or mutational load (Bertorelle et al., 2022; Caballero et al., 2017; Kardos et al., 2021; Robinson et al., 2023). Populations characterized by inbreeding depression typically show certain individual‐specific characteristics such as reproductive failure, but to what degree a population experiences inbreeding depression will depend on the strength of random genetic drift and the proportion of fixed deleterious mutations (i.e., drift load) relative to all individuals within the population (Hedrick & Kalinowski, 2000; Van Oosterhout, 2020).

Ultimately, the mutational load that a population (or species/subspecies) possesses will depend on its demographic history (Bertorelle et al., 2022; Robinson et al., 2023; Van der Valk et al., 2021). Species or subspecies with large effective population size (*N*_e_) tend to have higher mutational load in the form of inbreeding load compared to those with long‐term small *N*_e_. Because deleterious alleles are relatively rare within populations with large *N*_e_, they are less frequently expressed and remain as heterozygotes allowing the population to maintain its mutational load assuming random mating. In contrast, those populations that have existed at small *N*_e_ where inbreeding has been more common for long periods of time have had the opportunity to purge their inbreeding load by exposing deleterious alleles to selection and thereby reduce their frequency within the population. However, this pattern is based on only those populations that have survived and therefore currently observable, with an unknown number of other small undocumented populations having gone extinct (Soulé, 1987; see also Kardos et al., 2021). Therefore, caution is warranted before making any strong inferences concerning extinction risk based solely on standing genetic variation and inbreeding levels alone.

Similarly, we would be remiss to assume that genetic variation is not important in allowing populations to adapt to a changing environment (e.g., climate change, novel disease, and pathogens), and undoubtedly long‐term persistence will depend on their ability to meet those challenges (Foden et al., 2013; Lai et al., 2019). Our interest here is to highlight that additional measures are also needed along with quantifying genomic diversity to assess extinction risk, particularly among species or subspecies that have existed at small *N*_e_ for long periods of time, and determine if increasing genetic diversity is the most cost‐effective approach from a management perspective given that other factors may pose a more immediate threat toward a population's ability to persist (Jamieson, 2007; Teixeira & Huber, 2021; Yates et al., 2019).

Despite possessing significantly lower genomic diversity and higher inbreeding levels, island and nonmigratory peregrine subspecies each as a group do not appear to be at increased risk of future inbreeding depression relative to mainland and migratory subspecies given similar overall levels of inbreeding load (Figure 6c,d). However, because variation in inbreeding load does exist among and within specific subspecies or populations (Figure S4c,d) with some also possessing relatively high drift load (Figure S4a,b), management recommendations would benefit from identifying factors likely limiting population persistence at the local scale in addition to those associated with low genetic diversity or adaptation. For example, the island endemic subspecies, *F. p. nesiotes*, has an extremely small contemporary population size with most likely less than 150 breeding pairs on Fiji and Vanuatu (White, Cade, & Enderson, 2013) and possibly no more than 500 pairs in total when including the neighboring islands of New Caledonia and the Loyalty Islands (Baudat‐Francheschi et al., 2013). In agreement with previous studies investigating genetic diversity using nuclear microsatellite and mtDNA control region sequence data (Talbot et al., 2011; White, Sonsthagen, et al., 2013), our results indicate that this subspecies possessed extremely low genomic diversity.

Based on samples collected from Fiji and Vanuatu, *F. p. nesiotes* had over 5× lower heterozygosity (mean ± SD; *h* = 0.029 ± 0.015) and almost 4× the level of inbreeding (*F*_UNI_ = 0.525 ± 0.063) compared to the average estimate from mainland subspecies (*h* = 0.152 ± 0.037; *F*_UNI_ = 0.141 ± 0.114; Figure 4). Similarly, over 65% and 90% of ROH segments were ≥ 100 kb for Vanuatu and Fiji, respectively (*n* = 2 for each population), with the latter population possessing a much higher proportion of segments ≥2 Mb (Figure S3). These results suggest not only long‐term small *N*_e_ for the subspecies, but also recent decline on Fiji (Ceballos et al., 2018; Pemberton et al., 2012; Von Seth et al., 2021) that is consistent with evidence of inbreeding depression observed among a subset of local peregrines in the form of reproductive failure (Talbot et al., 2011). A similar pattern based on the proportion of ROH segment has also been observed with critically endangered kākāpō (*Strigops habroptilus*) on Steward Island in New Zealand compared to its mainland populations following recent decline due to introduced predators (Dussex et al., 2021). Increasing human population growth, especially on Fiji, may affect habitat and abundance of prey species and negatively impact the ability of *F. p. nesiotes* to persist (Talbot et al., 2011; White, Cade, & Enderson, 2013). Our results indicate that the subspecies is unique and further differentiated between the two islands (Figure 3a); thus, efforts are warranted to prevent any further decline by minimizing human‐related impacts. A similar concern is justified for island populations on Cape Verde (*F. p. madens*) and Iwo Islands (*F. p. furuitii*), as both subspecies possess a relatively high proportion of ROH segments ≥2 Mb in size (Figure S3) and high drift load in the form of homozygous‐derived deleterious genotypes (Figure S4a,b). Unfortunately, conservation efforts to prevent the extinction of *F. p. furuitii* may be too late because no individuals have not been seen or collected since 1938 (White, Cade, & Enderson, 2013).

Of the subspecies sampled that include both island and mainland populations (*F. p. pealei*, *F. p. cassini*, *F. p. peregrinator*, and *F. p. pelegrinoides*), individuals sampled from islands generally had reduced heterozygosity (Figure S2a) and increased inbreeding depression (Figure S2b) consistent with lower *N*_e_ as expected, but the proportion of segments in ROH were not necessarily higher with island populations compared to mainland populations. One exception was with *F. p. cassini*, whose mainland population in Chile had similar levels of ROH compared to its island counterpart in the Falklands (Figure S3). In fact, despite occupying a large geographic area throughout western and southern South America, mainland *F. p. cassini* had the highest inbreeding coefficient (*F*_UNI_ = 0.396 ± 0.041) and lowest heterozygosity (*h* = 0.057 ± 0.007) compared to all other mainland populations (see Figure S2). With a high proportion of ROH segments ≤2 Mb in length (Figure S3) and a relatively low long‐term PSMC‐inferred *N*_e_ (Figure S5), the results suggest that both island and mainland *F. p. cassini* populations have been at small *N*_e_ for many generations similar to sampled peregrine island populations. Given the extreme variation in habitat that exists from southwestern Colombia to southern Chile and Argentina (White, Cade, & Enderson, 2013), population differentiation may exist within the subspecies throughout South America.

Because we observed a similar pattern in genomic diversity levels between island and nonmigratory subspecies or populations, it is important to highlight that some mainland populations, in addition to *F. p. cassini*, have also persisted for many generations at small *N*_e_ with relatively low genomic diversity (e.g., *F. p. macropus*). In general, avian natal dispersal distances tend to be greater in migratory species than in closely‐related resident species (Newton, 2008), and therefore, the degree of population genetic differentiation and reduced *N*_e_ should be more pronounced within species or subspecies that possess reduced migratory behavior (Hung et al., 2017), but could also include populations that possess differing migration pathways or routes (Bensch, 1999; Böhning‐Gaese et al., 1998; Toews, 2017). Few studies, however, acknowledge that differences may exist at the intraspecific level based on migratory behavior. Nevertheless, the peregrine falcon may be a unique case with its large number of subspecies distributed globally, but the variation that exists among subspecies with migratory behavior could be an important factor broadly influencing genomic variation and their allelic composition among populations depending on dispersal patterns and their potential for range expansion (see also Gu et al., 2021).

Considerable overlap was observed among subspecies classified as either island or mainland and migratory or nonmigratory subspecies, rendering it difficult to discern which life‐history trait may influence genomic diversity specifically. Of the nine mainland subspecies that we were able to classify as possessing either migratory or nonmigratory behavior, only three were non‐migratory. Of those three nonmigratory mainland subspecies, both Australian subspecies, *F. p. submelanogenys* and *F. p. macropus*, had similar levels of heterozygosity, inbreeding coefficients, and inferred long‐term *N*_e_ as observed among island subspecies, whereas the Barbary falcon (*F. p. pelegrinoides*) from Israel had levels similar to other sampled migratory mainland subspecies (e.g., *F. p. tundrius*, *F. p. peregrinus*, and *F. p. calidus*).

The Barbary falcon has a fairly broad but fragmented distribution from the Canary Islands along northern Africa into the Sahel and the Middle East (Figure 2; White, Cade, & Enderson, 2013) with sampled populations possessing differing levels of genomic diversity. As mentioned previously, the subspecies' population on Canary Islands possessed reduced heterozygosity and increased inbreeding coefficients compared to its mainland counterpart (Figure S2), including a higher proportion of ROH segments ≥2 Mb in size (Figure S3), indicating fairly recent inbreeding effects. This pattern is consistent with the subspecies' long‐term *N*_e_ being similar between its island and mainland populations and likely a reflection of their recent shared ancestry. While Australian peregrines (i.e., *F. p. submelanogenys* and *F. p. macropus*) are thought to be fairly common ranging from 3000 to 5000 pairs (White, Cade, & Enderson, 2013), our estimates of genomic diversity and *N*_e_ are more characteristic of island subspecies. Similar to *F. p. cassini* in South America, these results suggest that population differentiation likely exists within Australia, and additional study is warranted using a much larger sample size.

Interestingly, even though genomic diversity was higher in migrant compared to nonmigrant subspecies, much less variation in total genomic diversity was observed among individuals identified as migrants (Figure 4). This was likely due to migrant peregrine subspecies occupying much larger geographic ranges with increased gene flow, or panmixia, and a greater opportunity to randomly share alleles resulting in increased genomic diversity than other isolated and geographically restricted mainland subspecies (Ortego et al., 2008). This pattern is further supported by higher long‐term *N*_e_ (Figure 7). In contrast, much more variation in genomic diversity existed among individuals that were nonmigratory mainland subspecies or those of unknown migratory status further highlighting that other factors such as spatial constraints and independent population histories within mainland distributions likely influence levels of genomic diversity differently within and among those populations.

Regardless of the cause, our results support theoretical expectations that long‐term *N*_e_ estimates among peregrine falcon subspecies were strongly correlated with genomic diversity (Figure S6). Whether the pattern was influenced more by spatial constraints or migratory behavior is inconsequential since the two traits are not mutually exclusive when subjected to comparison among peregrine subspecies. In a study comparing range size differences among North American passerine species, latitude was the strongest predictor explaining range size variation, whereas migratory status and distance traveled had no significant contribution toward explaining such patterns when controlling for latitude (Pegan & Winger, 2020). All of the peregrine subspecies that possess migratory behavior have distributions that extend over broad geographic areas within northern North America and Eurasia with low differentiation observed among neighboring subspecies (Figure 3a). While Gu et al. (2021) documented population‐level haplotype differences among Arctic Eurasian peregrines based on ADCY8, a gene shown to be associated with differences in migratory distance, our results indicated no differences in diversity levels at the whole‐genome level while using a subset of the same samples. The large geographic distributions among the migratory subspecies, and hence their large *N*_e_, therefore, allow for higher overall whole‐genome diversity than observed among most of the remaining peregrine subspecies.

In this study, we documented significant variation in genomic diversity among peregrine falcon subspecies and populations, which was strongly correlated with their estimates of long‐term *N*_e_ (Figure S6), yet measures associated with the mutational load varied with drift load (or homozygous deleterious variants) and inbreeding load (or heterozygous deleterious variants) showing contrasting correlations with *N*_e_ (Figure S7). In general, this could suggest that some populations or subspecies may have higher inbreeding tolerance given their demographic history and associated life histories (Robinson et al., 2023), but concern is warranted that a few subspecies also show signs of elevated drift load and its potential negative impact on fitness (e.g., *F. p. nesiotes*; Van Oosterhout, 2020). It could be generally assumed that geographically restricted and low dispersal taxa, or at least those that still persist, may tolerate ongoing inbreeding more than those that are widespread and experience high rates of gene flow due to overall differences relative to mutational load. However, processes unique to each population or lineage can undoubtedly influence its trajectory concerning their continued persistence depending on both random fluctuations associated with drift or factors influenced by the strength of selection (Kyriazis et al., 2023).

While genetic diversity is strongly linked to a populations' adaptive potential, our data focused on the peregrine falcon show that the extent of available genomic diversity that a population may possess is relative to its demographic history. In fact, it has been shown that peregrine falcons, including other large *Falco* species in general, have low intra‐ and interspecific variability at major histocompatibility complex (MHC) genes, which in most vertebrates is highly polymorphic due to balancing selection and its important role in mounting an adaptive immune response to exposed pathogens (Gangoso et al., 2012). It is not known if other compensatory mechanisms exist that function similar to an MHC immune response among large *Falco* species, yet the results from Gangoso et al. (2012) do suggest, however, that the maintenance of reduced MHC polymorphism has likely persisted for millions of years due to its shared ancestry across multiple falcon species. To what degree a lack of polymorphism exists among additional genes or gene complexes thought to have an important role in adaptive response within peregrine falcon deserves further study. The findings of this study therefore highlight that high levels of inbreeding may not necessarily translate to a lower likelihood of persistence due to inbreeding depression if that population has long‐term history of persisting at small size (Robinson et al., 2022). Thus, additional factors such as focusing priority on protecting habitat or reducing human persecution may be equally if not more important than increasing genetic diversity (Yates et al., 2019) when considering appropriate management decisions for species or subspecies with a similar demographic history of long‐term small population size.

## AUTHOR CONTRIBUTIONS

**Jeff A. Johnson:** Conceptualization (lead); data curation (lead); formal analysis (lead); funding acquisition (equal); investigation (lead); methodology (lead); project administration (lead); resources (lead); software (lead); supervision (lead); validation (lead); visualization (lead); writing – original draft (lead); writing – review and editing (lead). **Giridhar Athrey:** Data curation (supporting); formal analysis (equal); methodology (equal); software (supporting); validation (equal); writing – review and editing (supporting). **Clifford M. Anderson:** Conceptualization (supporting); funding acquisition (equal); investigation (supporting); resources (equal); writing – review and editing (supporting). **Douglas A. Bell:** Resources (equal); writing – review and editing (supporting). **Andrew Dixon:** Resources (equal); writing – review and editing (supporting). **Yoshinori Kumazawa:** Resources (supporting); writing – review and editing (supporting). **Tom Maechtle:** Resources (equal). **Garrett W. Meeks:** Investigation (supporting); methodology (supporting); writing – review and editing (supporting). **David Mindell:** Resources (supporting); writing – review and editing (supporting). **Keiya Nakajima:** Funding acquisition (supporting); resources (equal); writing – review and editing (supporting). **Ben Novak:** Conceptualization (equal); funding acquisition (equal); investigation (supporting); project administration (supporting); resources (supporting); writing – review and editing (supporting). **Sandra Talbot:** Resources (equal); writing – review and editing (supporting). **Clayton White:** Resources (equal); writing – review and editing (supporting). **Xiangjiang Zhan:** Resources (supporting); writing – review and editing (supporting).

## CONFLICT OF INTEREST STATEMENT

The authors declare no conflict of interest.

## BENEFIT‐SHARING STATEMENT

A research collaboration was created with scientists from countries providing samples for this study and all collaborators are included as co‐authors. This research addresses a priority concern by being the first study to include genomic samples from all named peregrine falcon subspecies with results on their evolutionary relationships.

## Supporting information


Data S1.

## Data Availability

Raw whole‐genome resequencing reads are available on the NCBI Short Read Archive (BioProject: accession no. PRJNA979930). Additional data that support the findings of this study are available in the Supporting Information of this article or available from the corresponding author upon reasonable request.
