# Supplementary material for: Whole‐genome survey reveals extensive variation in genetic diversity and inbreeding levels among peregrine falcon subspecies
Source: Ecol Evol. 2023 Jul 20;13(7):e10347. doi: 10.1002/ece3.10347 (PMC10361364; doi:10.1002/ece3.10347)
Supplement: Supplementary file 1 — Data S1. [file ECE3-13-e10347-s001.pdf]

SUPPLEMENTAL MATERIAL
for

Whole genome survey reveals extensive variation in genetic diversity and inbreeding levels among Peregrine Falcon subspecies

Jeff A. Johnson[1,2,*], Giridhar Athrey[3], Clifford M. Anderson[4], Douglas A. Bell[5,6], Andrew Dixon[7,8], Yoshinori Kumazawa[9], Tom Maechtle[10, †], Garrett W. Meeks[1], David Mindell[11], Keiya Nakajima[9,12], Ben Novak[13], Sandra Talbot[14], Clayton White[15], Xiangjiang Zhan[16]

Table S1. Sample list for all named 19 Peregrine Falcon subspecies and three outgroup taxa.

Figure S1. MapDamage characterization of damage patterns in genomic DNA from Peregrine Falcon museum toe-pad tissue samples.

Figure S2. Mean variation in the proportion of SNPs heterozygous and inbreeding coefficient for Peregrine Falcon subspecies and populations.

Figure S3. The mean proportion of genomic segments in ROH among Peregrine Falcon subspecies and populations.

Figure S4. Mean proportion of homozygous-derived and heterozygous HIGH Impact and LOF variants among Peregrine Falcon subspecies and populations.

Figure S5. Mean effective population size (N_e) over the past ~1 million years for Peregrine Falcon subspecies based on autosomal SNPs using PSMC.

Figure S6. Mean effective population size (N_e) correlations with proportion of SNPs heterozygous, inbreeding coefficient (F_{UNI}), and % of ROH segments ≥ 100kb and 2Mb.

Figure S7. Mean effective population size (N_e) correlations with proportion of homozygous-derived and heterozygous HIGH Impact and LOF variants observed among migrant & non-migrant and mainland & island populations.

Table S1. Sample list for all 19 Peregrine Falcon subspecies and three outgroup taxa. Average depth of coverage and % missing calls after filtering and categories used for each sample for comparing diversity metrics. Subspecies without migratory status indicated were excluded in those analyses due to uncertainty in their classification.

Taxon	Location sampled (sample ID)	Avg depth	% missing calls	Realm	Migratory status	Mainland or Island
F. p. anatum	Alaska, Tanana River, USA (A42.S42)	12.9	0.0039	Nearctic	migratory	mainland
F. p. anatum	Alberta, Rosebud River, Canada (JJ014_S41)	13.7	0.0031	Nearctic	migratory	mainland
F. p. anatum	California, San Fransisco, USA (JJ033_S43)	12.0	0.0043	Nearctic	migratory	mainland
F. p. anatum†	Colorado, USA (CU-12)	1.1	NA	Nearctic	migratory	mainland
F. p. anatum	Oregon, Curry County, USA (JJ012_S40)	12.1	0.0055	Nearctic	migratory	mainland
F. p. babylonicus	Ömnögovi, Mongolia (1.3)	22.4	0.0018	Palearctic		mainland
F. p. babylonicus	Ömnögovi, Mongolia (2.3)	19.5	0.0020	Palearctic		mainland
F. p. babylonicus	Kazakhstan (JJ008_S28)	10.1	0.0074	Palearctic		mainland
F. p. babylonicus	UAE, Dubai (Falco5_S27)	11.0	0.0060	Palearctic		mainland
F. p. brookei	Spain, Huelva (JJ016_S34)	12.5	0.0044	Palearctic		mainland
F. p. brookei	Spain, Cadiz (JJ019_S35)	10.8	0.0060	Palearctic		mainland
F. p. brookei	unknown (Falco54_S36)	10.4	0.0071	Palearctic		mainland
F. p. calidus	Russia, Yamal Peninsula (Yamal1)‡	29.4	0.0018	Palearctic	migratory	mainland
F. p. calidus	Russia, Yamal Peninsula (Yamal2)‡	31.7	0.0021	Palearctic	migratory	mainland
F. p. calidus	Russia, Yamal Peninsula (Yamal5)‡	30.8	0.0023	Palearctic	migratory	mainland
F. p. calidus	Russia, Yamal Peninsula (Yamal6)‡	31.4	0.0017	Palearctic	migratory	mainland
F. p. calidus	Russia, Yamal Peninsula (Yamal7)‡	32.3	0.0019	Palearctic	migratory	mainland
F. p. calidus	Russia, Yamal Peninsula (Yamal8)‡	34.5	0.0019	Palearctic	migratory	mainland
F. p. calidus	Russia, Yamal Peninsula (Yamal9)‡	30.3	0.0017	Palearctic	migratory	mainland
F. p. calidus	Russia, Yamal Peninsula (Yamal10)‡	28.1	0.0018	Palearctic	migratory	mainland
F. p. calidus	Russia, Yamal Peninsula (Yamal11)‡	30.3	0.0018	Palearctic	migratory	mainland
F. p. cassini	Chile, Straits of Magellen (PEFABA131_S11)	9.7	0.0091	Nearctic		mainland

F. p. cassini	Chile, Straits of Magellen (PEFABA72_S7)	10.9	0.0064	Nearctic		mainland
F. p. cassini	Chile, Straits of Magellen (PEFABA82_S8)	9.4	0.0095	Nearctic		mainland
F. p. cassini	East Falkland Island (PEFA111_S10)	11.0	0.0062	Nearctic		island
F. p. cassini	East Falkland Islands (PEFABA910_S9)	10.3	0.0074	Nearctic		island
F. p. ernesti	New Guinea (AMNH 537359; BN137)[§]	8.1	0.0403	Australasian	non-migratory	island
F. p. ernesti	New Guinea (AMNH 791024; BN140)[§]	8.3	0.0623	Australasian	non-migratory	island
F. p. ernesti	New Guinea, Southeast (AMNH 537362; BN142)[§]	11.6	0.0120	Australasian	non-migratory	island
F. p. furuitii	Iwoto, Japan (AMNH 470357; BN135)[§]	7.1	0.0583	Palearctic	non-migratory	island
F. p. furuitii	Iwoto, Japan (AMNH 470358; BN136)[§]	7.0	0.0523	Palearctic	non-migratory	island
F. p. harterti	Russia, Kolyma River (Kolyma1)[‡]	30.2	0.0022	Palearctic	migratory	mainland
F. p. harterti	Russia, Kolyma River (Kolyma4)[‡]	34.2	0.0022	Palearctic	migratory	mainland
F. p. harterti	Russia, Kolyma River (Kolyma5)[‡]	35.6	0.0023	Palearctic	migratory	mainland
F. p. harterti	Russia, Kolyma River (Kolyma6)[‡]	29.8	0.0021	Palearctic	migratory	mainland
F. p. harterti	Russia, Kolyma River (Kolyma7)[‡]	34.0	0.0022	Palearctic	migratory	mainland
F. p. harterti	Russia, Kolyma River (Kolyma8)[‡]	32.3	0.0021	Palearctic	migratory	mainland
F. p. harterti	Russia, Kolyma River (Kolyma9)[‡]	30.2	0.0021	Palearctic	migratory	mainland
F. p. japonensis	Japan (Fpj-4)	4.5	0.3183	Palearctic	non-migratory	island
F. p. japonensis	Japan (Fpj-5)	4.6	0.2933	Palearctic	non-migratory	island
F. p. macropus	Australia (R0002515, FALCO37_S21)	10.1	0.0095	Australasian	non-migratory	mainland
F. p. macropus	Australia, Victoria (PEFAVIC205_S19)	10.4	0.0073	Australasian	non-migratory	mainland
F. p. macropus†	Australia, Victoria (PEFAVIC206_S20)	2.0	NA	Australasian	non-migratory	mainland
F. p. madens	Cape Verde (CV3B_S5)	10.6	0.0045	Afrotropical	non-migratory	island
F. p. madens	Cape Verde (CV3B_S6)	11.0	0.0055	Afrotropical	non-migratory	island
F. p. minor	South Africa (JJ021_S37)	11.9	0.0049	Afrotropical		mainland
F. p. minor	Zimbabwe, Harare (JJ024_S38)	11.1	0.0062	Afrotropical		mainland

F. p. minor	Zimbabwe, Harare (JJ025_S39)	11.9	0.0065	Afrotropical		mainland
F. p. nesiotes	Fiji (102_S13)	12.3	0.0037	Australasian	non-migratory	island
F. p. nesiotes	Fiji (Fp16_S12)	10.5	0.0060	Australasian	non-migratory	island
F. p. nesiotes	Vanuatu, Tukutuku (Fp14_S15)	10.5	0.0073	Australasian	non-migratory	island
F. p. nesiotes	Vanuatu, Erromango (VAN117_S14)	8.5	0.0150	Australasian	non-migratory	island
F. p. pealei	Alaska, Amchitka Island, USA (CMW2-108_S16)	11.1	0.0056	Nearctic		island
F. p. pealei	Alaska, Buldir Island, USA (BUL2B_S17)	10.8	0.0061	Nearctic		island
F. p. pealei	Alaska, Klokachef Island, USA (987-64102_JJ037_S18)	9.6	0.1585	Nearctic		mainland
F. p. pelegrinoides	Canary Islands, Fuerteventura (FPELC03FUE_S22)	11.0	0.0059	Palearctic	non-migratory	island
F. p. pelegrinoides	Canary Islands, Lanzarote (FPELC03LANZ_S23)	8.2	0.0186	Palearctic	non-migratory	island
F. p. pelegrinoides	Israel (I-1_S24)	10.7	0.0073	Palearctic	non-migratory	mainland
F. p. pelegrinoides	Israel (I-2_S25)	12.5	0.0046	Palearctic	non-migratory	mainland
F. p. pelegrinoides	Israel (I-6_S26)	11.2	0.0054	Palearctic	non-migratory	mainland
F. p. peregrinator	China, Foochow (AMNH 537350; BN144)[§]	9.7	0.0215	Indomalayan	non-migratory	mainland
F. p. peregrinator	China, Foochow (AMNH 537351; BN145)[§]	7.3	0.0561	Indomalayan	non-migratory	mainland
F. p. peregrinator	India (NMWG599; JJ011)	4.6	0.2216	Indomalayan	non-migratory	mainland
F. p. peregrinator	Sri Lanka (3934_S3)	11.3	0.0060	Indomalayan	non-migratory	island
F. p. peregrinator	unknown (Falco8_S4)	11.9	0.0050	Indomalayan	non-migratory	
F. p. peregrinus	Russia, Kola Peninsula (Kola1)[‡]	30.6	0.0022	Palearctic	migratory	mainland
F. p. peregrinus	Russia, Kola Peninsula (Kola2)[‡]	31.0	0.0022	Palearctic	migratory	mainland
F. p. peregrinus	Russia, Kola Peninsula (Kola4)[‡]	30.2	0.0021	Palearctic	migratory	mainland
F. p. peregrinus	Russia, Kola Peninsula (Kola5)[‡]	33.1	0.0019	Palearctic	migratory	mainland
F. p. peregrinus	Russia, Kola Peninsula (Kola6)[‡]	34.3	0.0023	Palearctic	migratory	mainland
F. p. peregrinus	Russia, Kola Peninsula (Kola7)[‡]	32.4	0.0019	Palearctic	migratory	mainland
F. p. peregrinus	Russia, Kola Peninsula (Kola8)[‡]	31.9	0.0019	Palearctic	migratory	mainland
F. p. peregrinus	Russia, Kola Peninsula (Kola9)[‡]	34.3	0.0020	Palearctic	migratory	mainland

F. p. peregrinus	Russia, Kola Peninsula (Kola10)[‡]	32.2	0.0020	Palearctic	migratory	mainland
F. p. peregrinus	Russia, Kolguev Island (Kolguev1)[‡]	31.6	0.0021	Palearctic	migratory	mainland
F. p. peregrinus	Russia, Kolguev Island (Kolguev2)[‡]	32.6	0.0021	Palearctic	migratory	mainland
F. p. peregrinus	Russia, Kolguev Island (Kolguev3)[‡]	33.3	0.0024	Palearctic	migratory	mainland
F. p. peregrinus	Russia, Kolguev Island (Kolguev4)[‡]	32.9	0.0023	Palearctic	migratory	mainland
F. p. peregrinus	Russia, Kolguev Island (Kolguev5)[‡]	32.9	0.0019	Palearctic	migratory	mainland
F. p. peregrinus	Sweden, North (NMWG50; JJ003_S30)	11.8	0.0064	Palearctic	migratory	mainland
F. p. peregrinus	Sweden, North (NMWG56; JJ007_S32)	8.7	0.0160	Palearctic	migratory	mainland
F. p. peregrinus	Scotland (R0000271; Falco35_S33)	12.0	0.0049	Palearctic	migratory	mainland
F. p. peregrinus	Sweden, Southwest (NMWG54; JJ005_S31)	11.9	0.0054	Palearctic	migratory	mainland
F. p. radama	Madagascar (JJ001_S29)	10.1	0.0059	Afrotropical	non-migratory	island
F. p. submelanogenys	Australia, Southwest (Fps-1.3)	16.5	0.0102	Australasian	non-migratory	mainland
F. p. submelanogenys	Australia, Southwest (Fps-2.3)	15.8	0.0109	Australasian	non-migratory	mainland
F. p. submelanogenys	Australia, Southwest (Fps-3.2)	15.4	0.0018	Australasian	non-migratory	mainland
F. p. tundrius	Alaska, Colville River, USA (1807-00802; 1613_S47)	10.0	0.0085	Nearctic	migratory	mainland
F. p. tundrius	Greenland, Kangerlussuaq (3024027; JJ043_S49)	14.5	0.0032	Nearctic	migratory	mainland
F. p. tundrius	Greenland, Thule (30224009; TPF183_S46)	12.0	0.0051	Nearctic	migratory	mainland
F. p. tundrius	unknown, migrant (1947-29916; JJ002_S48)	11.2	0.0060	Nearctic	migratory	mainland
F. p. tundrius	NWT Territories, Rankin Inlet, Canada (SAS330; JJ029_S44)	12.9	0.0034	Nearctic	migratory	mainland
F. p. tundrius	Quebec, Ungava Bay, Canada (MG3; JJ031_S45)	11.4	0.0051	Nearctic	migratory	mainland
Falco mexicanus	California, Siskiyou County[¶]	16.6	0.0030	NA	NA	NA
Falco cherrug	unknown[⊕]	11.3	0.0322	NA	NA	NA
Falco fasciinucha	Africa (TF1)	4.3	0.3020	NA	NA	NA

Falco fasciinucha	Africa (TF2)	3.4	0.3970	NA	NA	NA

† samples not used in analyses due to low depth of coverage

‡ raw reads obtained from Genbank (Gu et al. 2021 Nature 591:259-264)

§ toe-pad tissues obtained from study skins at American Museum of Natural History (AMNH), New York, USA

¶ raw reads obtained from Genbank (see Doyle et al. 2018 BMC Genomics 19: 233)

⊕ raw reads obtained from Genbank (see Zhan et al. 2013 Nature Genetics 45:563-566)

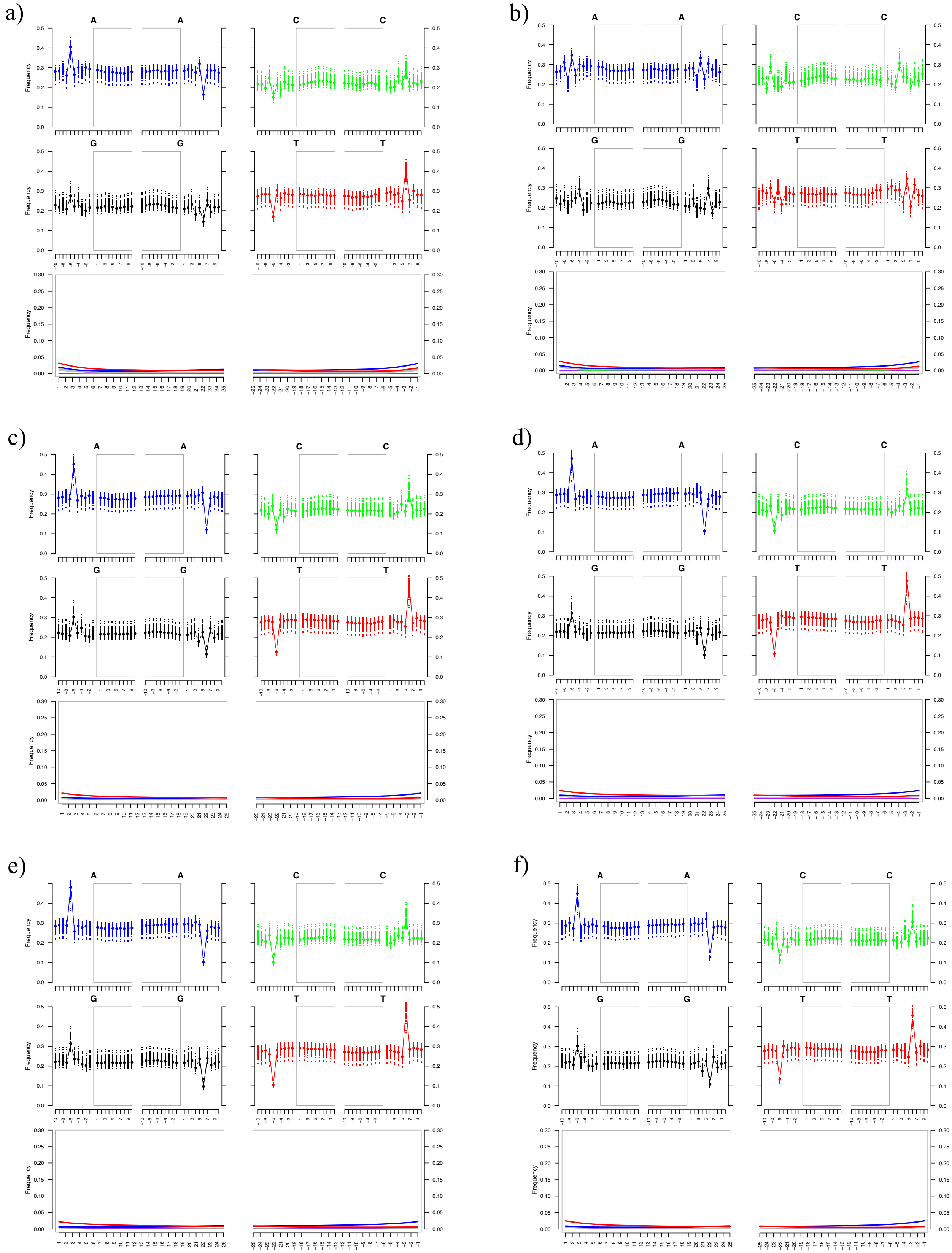
a)
b)
c)
d)
e)
f)
A
C
G
T
Frequency

g)

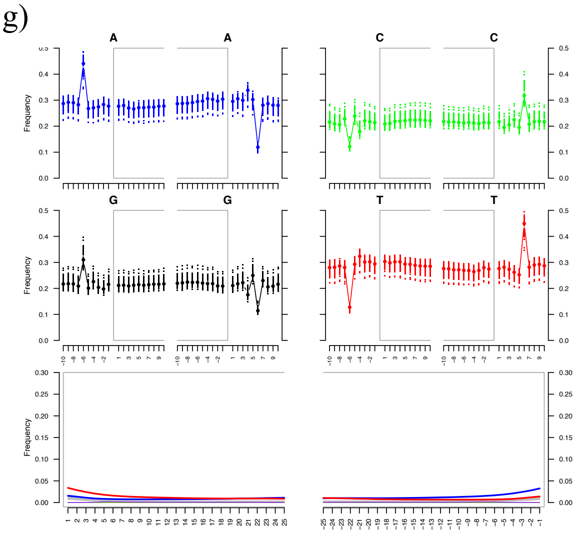

Figure S1. Characterization of damage patterns in genomic DNA from Peregrine Falcon museum toe-pad tissue samples (n=7) based on their mapping to reference genome. a) *F. p. ernesti* (BN137), b) *F. p. ernesti* (BN140), c) *F. p. ernesti* (BN142), d) *F. p. furuitii* (BN135), e) *F. p. furuitii* (BN136), f) *F. p. peregrinator* (BN144), and g) *F. p. peregrinator* (BN145). The increased frequency of purines (guanine, G; adenine, A) and pyramidines (cytosine, C; thymine, T) immediately upstream of the 5' and downstream of the 3' ends of reads, respectively, is consistent with depurination-induced fragmentation. The lower plots show an increasing proportion of cytosines that are deaminated toward the end reads (C to T substitutions: red, G to A substitutions: blue). All subsequent processing steps used BAM rescaled base quality scores as implemented using mapDamage with additional filtering steps applied to help mitigate the effects of DNA damage in the generated consensus sequences.

a)

b)

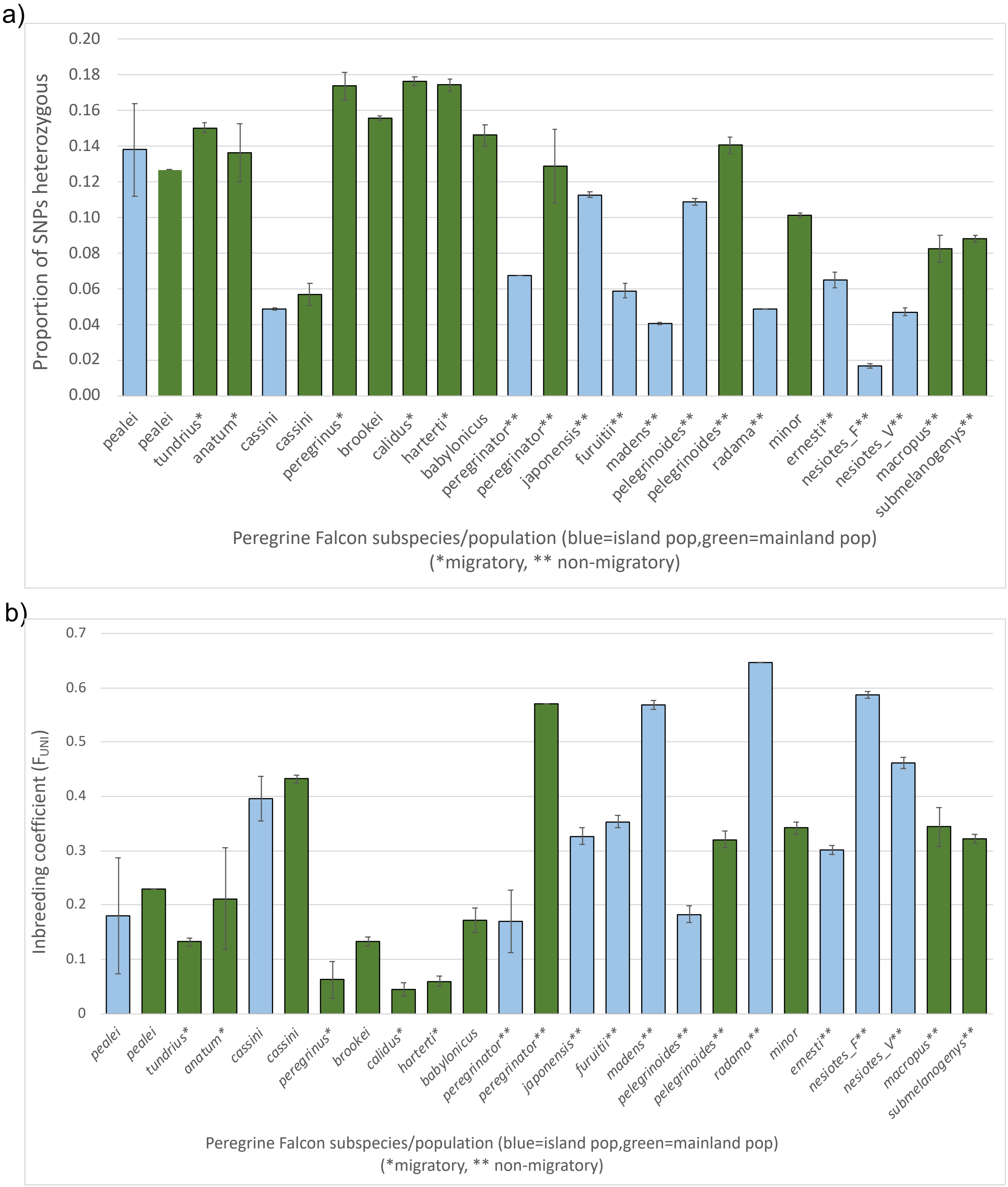

Figure S2. Mean variation in a) the proportion of SNPs heterozygous and b) inbreeding coefficient for Peregrine Falcon subspecies and populations using autosomal intergenic biallelic SNPs. Values for subspecies with both mainland and island-restricted populations (*F. p. pealei*, *F. p. cassini*, *F. p. peregrinator*, and *F. p. pelegrinoides*) are shown separately and those for *F. p. nesiotes* populations are shown by their island of origin (F, Fiji; V, Vanuatu). (mainland, green; island, blue; * migratory; ** non-migratory)

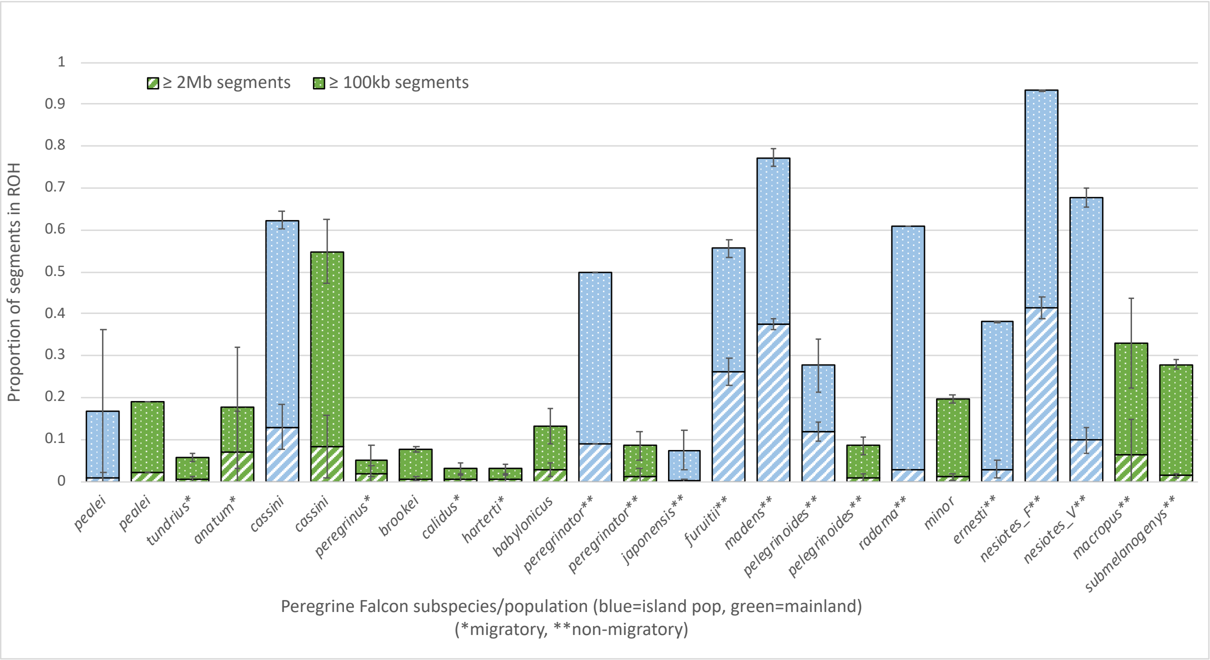

Figure S3. The mean proportion of genomic segments in ROH among Peregrine Falcon subspecies using genic and intergenic autosomal SNPs. Values for subspecies with both mainland and island-restricted populations (*F. p. pealei*, *F. p. cassini*, *F. p. peregrinator*, and *F. p. pelegrinoides*) are shown separately. (mainland, green; island, blue; * migratory; ** non-migratory).

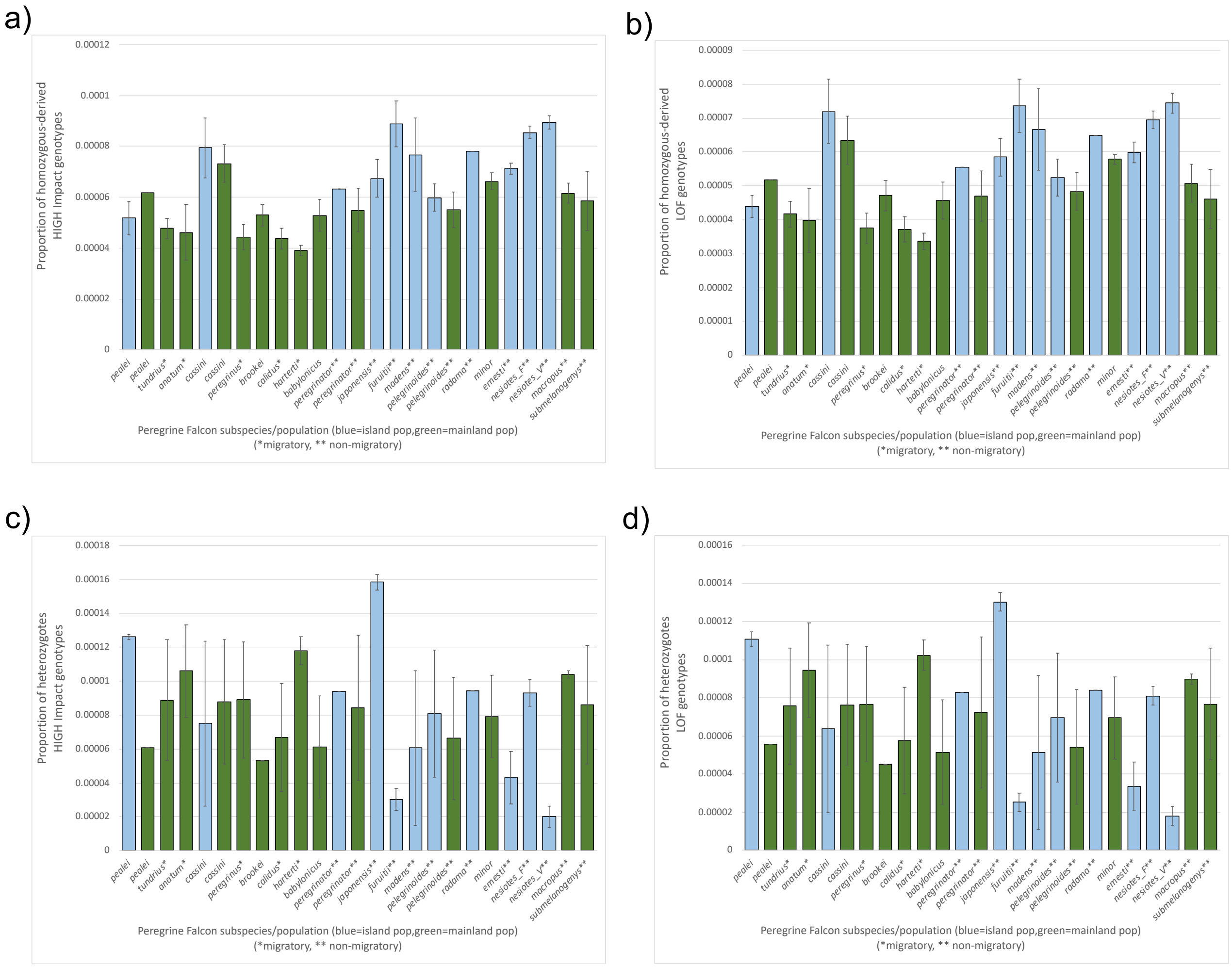

Figure S4. Mean proportion of (a, b) homozygous-derived and (c, d) heterozygous HIGH Impact and LOF variants among Peregrine Falcon subspecies and populations. Values for subspecies with both mainland and island-restricted populations (*F. p. pealei*, *F. p. cassini*, *F. p. peregrinator*, and *F. p. pelegrinoides*) are shown separately. (mainland, green; island, blue; * migratory; ** non-migratory).

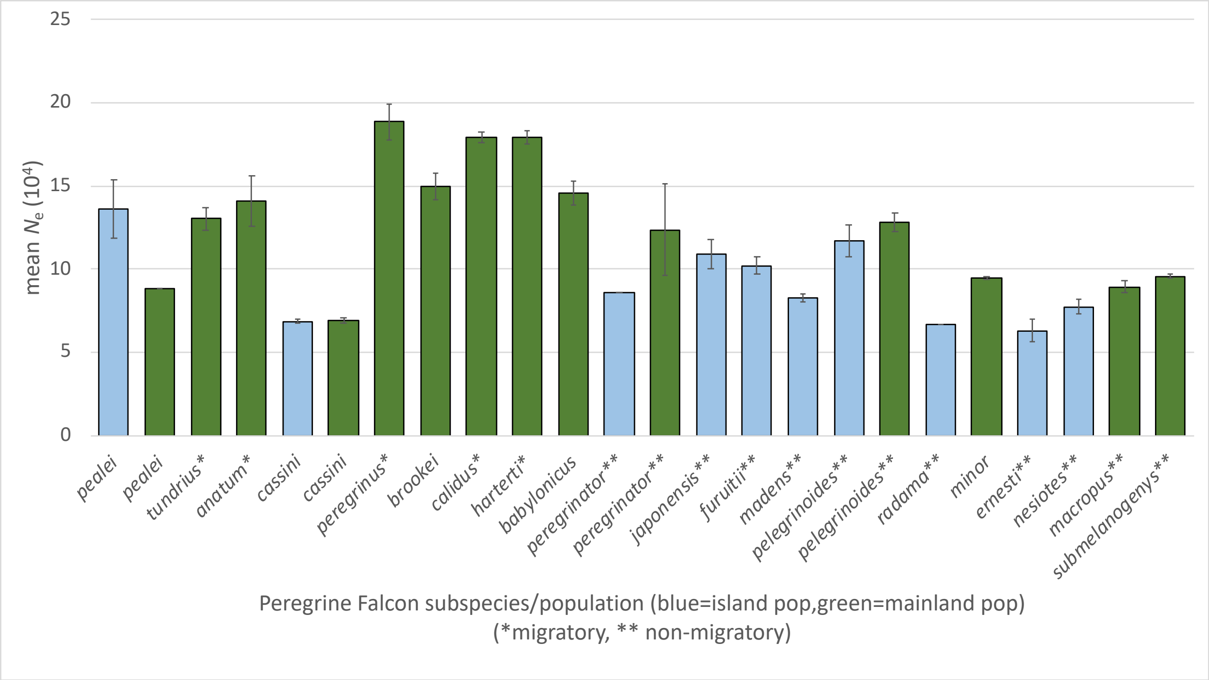

Figure S5. Mean effective population size (N_e) over the past ~1 million years for Peregrine Falcon subspecies based on autosomal SNPs using PSMC. Values for subspecies with both mainland and island-restricted populations (*F. p. pealei*, *F. p. cassini*, *F. p. peregrinator*, and *F. p. pelegrinoides*) are shown separately (mainland, green; island, blue; * migratory; ** non-migratory).

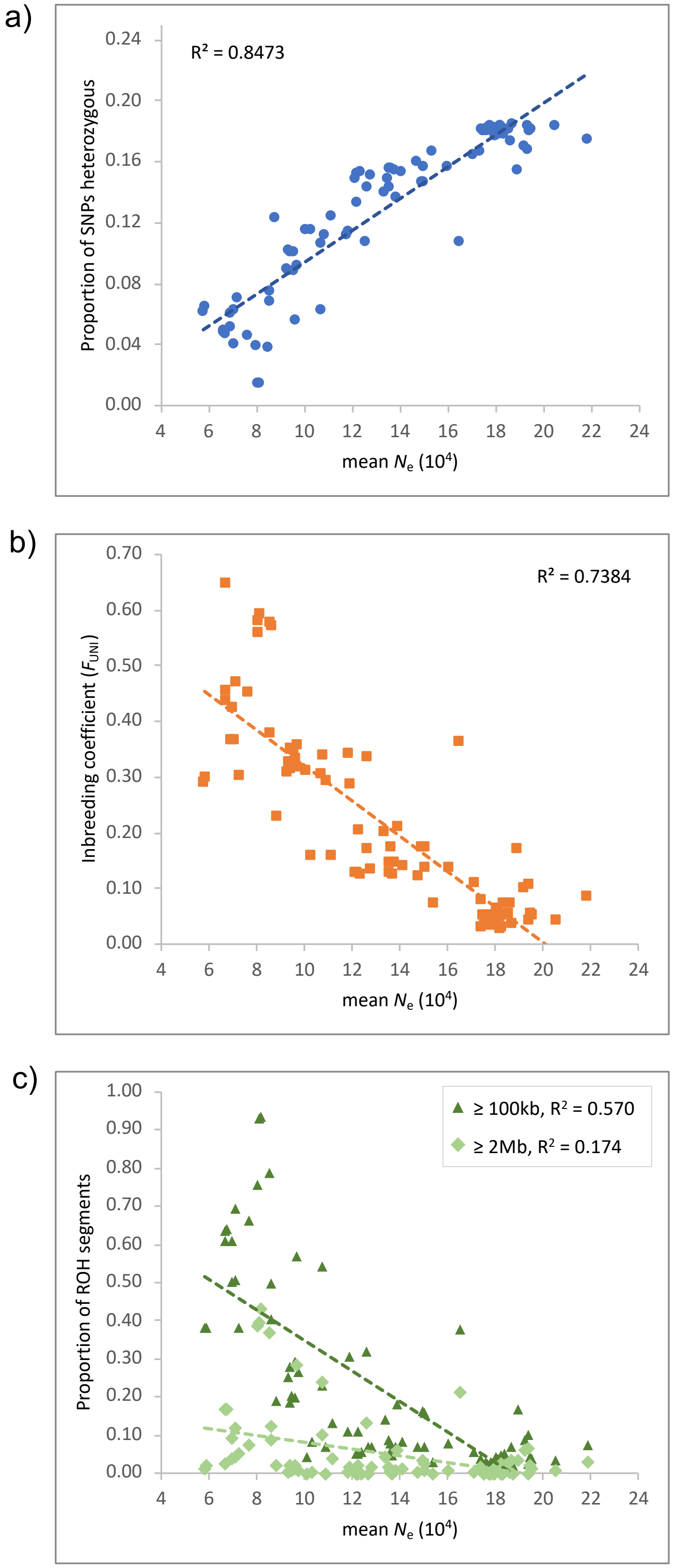

Figure S6. Mean effective population size (N_e) correlations with a) proportion of SNPs heterozygous, b) inbreeding coefficient (F_{UNI}), and c) % of ROH segments ≥ 100kb and 2Mb.

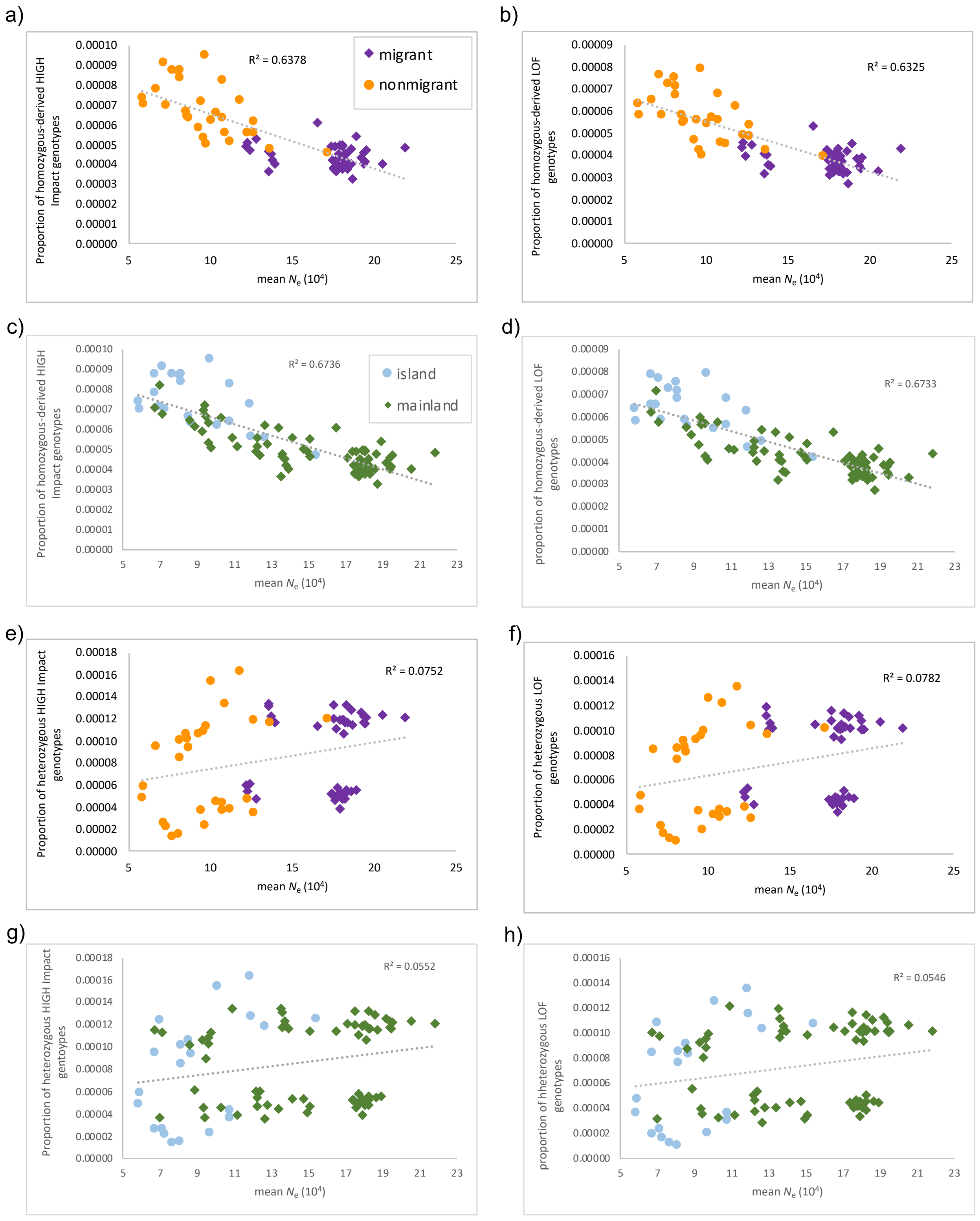

Figure S7. Mean effective population size (N_e) correlations with proportion of (a-d) homozygous-derived and (e-h) heterozygous HIGH Impact and LOF variants observed among migrant & non-migrant and mainland & island populations.
